# Tracking Small Microplastics and Plastic Additives in Freshwater: An Integrated Analytical Approach

**DOI:** 10.3390/molecules31213973

**Published:** 2026-11-04

**Authors:** Greta Palombella, Beatrice Rosso, Elisa Scalabrin, Roberta Zangrando, Andrea Gambaro, Fabiana Corami

**Affiliations:** 1 Department of Environmental Sciences, Informatics and Statistics, Ca’ Foscari University, via Torino 155, 30172 Venice, Italy; greta.palombella@unive.it (G.P.); beatrice.rosso@unive.it (B.R.); elisa.scalabrin@cnr.it (E.S.); roberta.zangrando@cnr.it (R.Z.); gambaro@unive.it (A.G.); 2 Institute of Polar Sciences—National Research Council (CNR-ISP), via Torino 155, 30172 Venice, Italy; 3 National Interuniversity Consortium for Environmental Sciences (CINSA), via Torino 155, 30172 Venice, Italy

**Keywords:** microplastic, small microplastics, plastic additive, drinking water safety, freshwater systems, HRMS, Micro-FTIR

## Abstract

Microplastic (MP) and plastic additive (PA) pollution in freshwater systems threatens drinking water safety. This study proposes an integrated multi-analytical approach to characterize small microplastics (SMPs < 100 μm), PAs, and other microlitter components (MLCs) in a canal supplying a drinking water treatment plant. The method combines Micro-FTIR spectroscopy for particulate SMP and MLC identification with HPLC-Orbitrap for Non-Targeted Screening (NTS) and Suspect Screening Analysis (SSA) of dissolved PAs. Results showed the highest SMP abundance in Autumn (10,116 SMPs L^−1^), with 20 polymer types identified. Polytetrafluoroethylene (PTFE) was most abundant, followed by high-density polyethylene (HDPE); tire wear particles (TWPs) were also detected. Micro-FTIR identified antistatic agents, fillers, and lubricants as the main particulate PAs. SMPs averaged 57 μm in length and 32 μm in width. HPLC-Orbitrap revealed a predominance of plasticizers and flame retardants, specifically diethyl phthalate (DEP), dibutyl phosphate (DBP), tributyl phosphate (TBP), and tris(2-chloroethyl) phosphate (TCEP). Although EU regulations highlight the need to monitor MPs, specific regulatory limits and standardized treatment removal efficiencies remain undefined. This comprehensive assessment of particulate and dissolved fractions establishes a methodological framework to guide future safety standards and optimize water treatment.

## 1. Introduction

Microplastics (MPs) are a highly heterogeneous class of particles ranging in size from 1 μm to 5 mm and occurring in various shapes, from spherical to elongated, including fibers and irregular fragments [1,2]. Based on their origin, MPs can be classified as primary, when they are intentionally manufactured at small sizes, or secondary, resulting from the fragmentation of larger plastic items (e.g., bottles, bags, fishing nets) due to UV radiation and mechanical forces [3]. Small microplastics (SMPs < 100 µm), which constitute the dominant environmental fraction [4], exhibit high mobility and are readily ingested by invertebrates, entering food webs and posing human health risks [5,6,7].

Over 13,000 chemicals are associated with plastics, comprising non-intentionally added substances—such as adsorbed polycyclic aromatic hydrocarbons, trace metals, and microorganisms [8,9] —and intentionally added Plastic Additives (PAs) [10]. PAs encompass diverse functional classes categorized by ECHA [11] as well as processing aids like lubricants and cross-linking agents. Among these, flame retardants, plasticizers, and fillers dominate market demand [12,13], accounting for ~75% of annual additive production [14]. A single plastic item can contain up to 20 additives, representing up to 70% of its total weight [15,16]. Because PAs possess variable binding interactions with the polymer matrix [17], they leach into aquatic environments at different rates, potentially exerting toxic effects or degrading into hazardous transformation products [13,18,19,20,21].

Together with non-plastic anthropogenic particles, such as natural and artificial fibers (e.g., cellulose and rayon or viscose, respectively) [22,23,24,25], PAs constitute the broader framework of micro-litter components (MLCs), which are evaluated and reported as distinct analytical categories throughout this study.

In recent years, MPs have been extensively investigated across a wide range of environmental and non-environmental matrices. Indeed, their presence has been reported not only in surface waters, but also in drinking water, including both tap and bottled water [26]. Global studies on tap water have reported a wide range of MP concentrations, including particles up to 5 mm [27]. In Europe specifically, several studies have detected varying concentrations within smaller size fractions, ranging from 0.2 μm to 379 μm [28,29,30]. The reported concentrations and size distributions are strongly influenced by the sampling methodology, the analytical instrumentation used, the operational efficiency and configuration of the drinking water treatment plant, and the characteristics of the raw water source feeding the plant [31,32,33].

Despite extensive investigation [26,27,28,29,30,31,32,33], MPs remain insufficiently regulated in drinking water. Directive (EU) 2020/2184 [34] provided a legal framework for MP monitoring, and Decision (EU) 2024/1441 [35] established a harmonized measurement methodology; however, enforceable threshold limits are still lacking. Furthermore, while polymer characterization has received significant attention, combined temporal assessments of MPs and their associated chemical additives across both particulate and dissolved phases remain scarce.

Given this evolving regulatory landscape and the “One Health” framework, generating high-resolution spatial and temporal datasets is critical to elucidate MP environmental fate and their relationship with PAs. This is especially needed for strategic drinking water resources exposed to multiple anthropogenic pressures. In this study, we investigated MPs and PAs in an artificial canal diverting water from the Sile River to the drinking water treatment plant serving Venice (Italy), a key surface water source vulnerable to regional land-use impacts. To this end, a full-year monthly monitoring campaign was conducted using Micro-FTIR to elucidate the temporal dynamics and polymer composition of MPs and MLCs. Complementarily, HPLC-HRMS non-target screening was applied across a three-month sampling window as a pilot proof-of-concept. This exploratory screening aims to test the feasibility of comprehensive PA profiling across particulate and dissolved phases, establishing a baseline to guide future extended monitoring. Using a novel multi-analytical approach, we demonstrate effective comprehensive profiling of PAs and show that ubiquitous, persistent PAs can serve as complementary indicators for tracing MP sources and transport pathways in aquatic systems.

## 2. Results

### 2.1. SMPs Quantification and Chemical Identification in Water Samples via Micro-FTIR

The SMP abundance (SMPs/L) with the respective measurement error in water samples is shown in Figure 1. Since TWPs have been recently considered as MPs [36,37,38], they were included in the total abundance of SMPs detected for all samples (Figure 1a). All typologies of polymers and their acronyms are listed in Table S3. A selection of the highest-quality spectra is shown in Figure S5.

SMPs total abundance (SMPs/L) was higher during the autumn and winter compared to spring and summer. Except for the month of June, this pattern was observed both when considering the total abundance of SMPs and also when TWPs were excluded (Figure 1b). Specifically, the lowest abundance was observed in April 2023 (2710 ± 72 SMPs/L) and the highest in October 2023 (10,116 ± 95 SMPs/L). Excluding TWPs, the sample abundance in June is comparable to that of October. The high abundance of SMPs in October could be correlated with weather conditions; specifically, September 2023 saw 55% less rainfall than average, while October recorded a 65% increase above the average rain regime [39]. This higher rainfall regime could have led to an increase in wet deposition and runoff. Indeed, in high-particulate-matter regions such as the Po Valley [40], atmospheric wet deposition and subsequent surface runoff represent highly plausible contributors to seasonal MP fluctuations in aquatic bodies [41,42]. TWPs constituted the most abundant category of SMPs, accounting for over 50% of the total in every month except June, with the highest 81% in July. The differences observed through the months were highly statistically significant, according to the Kruskal–Wallis test *p* ≤ 0.01.

Twenty different polymers (including TWPs) were optimally identified. This achievement is particularly noteworthy given that many studies in the literature often focus on a limited range of polymers due to their pretreatment method, overlooking high-density polymers (e.g., fluorinated polymers), and due to the analytical method (e.g., a limited set of particles analyzed) [43,44,45]. On the other hand, the oleo-extraction procedure [46] and the reference spectral libraries employed in the analysis allowed the identification of a wide range of SMPs. The most abundant polymer was PTFE, followed by HDPE and PA6. LDPE, EVA, PEAA-Zn, PES and others were identified in the lowest amount. While certain polymers may originate from more specific applications, other polymers are widely employed; therefore, source attribution may be challenging due to overlapping uses and environmental fragmentation processes [47].

The presence of various polymers in the samples from the irrigation canal might stem not only from direct inputs from urban and agricultural surroundings, but potentially also from atmospheric deposition [41,42], which is recognized as a potential vector for the diffuse transport of these pollutants across environments. The canal’s exposure to surrounding land uses, such as agricultural activities, could tentatively explain some of the observed contamination patterns. In this context, major environmental sources of PTFE might include abrasion or degradation from industrial gaskets, filtration membranes, and technical component wear, as well as potential agricultural uses such as tubing and pipelines.

HDPE and LDPE are both widely used in industrial, packaging, and agricultural contexts, particularly in irrigation tubing, mulch films, fertilizer bags, and packaging materials, and their presence likely reflects diffuse inputs from agricultural activities.

EVA, a copolymer commonly used in food and medical packaging, adhesives, coatings, shoes, mats, and padding materials, may also originate from degraded materials used in farming equipment or construction residues present in the catchment area [48,49,50]. PA6 is commonly used in textiles, but also munching films, ropes and fibers, mainly used in vineyards. Together with PA6, PES is a major synthetic fiber used in clothing and technical textiles, but it is also employed for supports and ties and as a material for agricultural structures and roofing. Its presence is frequently correlated with the atmospheric transport of airborne SMPs [51,52]. These findings could reflect a potential combination of point and diffuse sources, which would be broadly aligned with the multifunctional use of the surrounding urban centers, agricultural land, waste and potential atmospheric depositions [48,53].

Across all water samples, average microplastic (MP) size was 57 µm (length) by 32 µm (width). Although maximum sizes reached 679 µm, most particles fell into the Small Microplastic (SMP) category (<100 µm, Figure S6), predominantly ranging within 21 µm, with minor peaks at 56–76 µm and 80–95 µm.

### 2.2. MLCs Quantification and Chemical Identification in Water Samples via Micro-FTIR

The MLC category encompasses non-plastic anthropogenic debris, including natural and semi-synthetic non-plastic fibers, alongside PAs identified on particle surfaces through Micro-FTIR analysis. The MLC abundance (MLCs/L) with the respective Poisson error confidence limit (measurement error) in water samples is shown in Figure 2. In Figure 2a, all identified individual MLCs were shown separately; while in Figure 2b, the same data are aggregated into broader categorical classes. As for MPs, it is important to note that many of these compounds are employed in multiple applications; however, due to the difficulty in identifying specific use categories, only their primary use has been considered. The trend for MLCs is less pronounced compared to the SMPs one; however, a general pattern of increase during the colder seasons can still be identified, starting from October (with a maximum peak of 18,065 ± 189 MLCs/L) and extending through January. Antistatic agents and natural fibers are the most abundant categories, followed by fillers, lubricants, and plasticizers (Table S3).

Considering all water samples, the average size of MLCs was very similar to SMPs: 59 µm for length and 38 µm for width. The highest concentration of MLCs is found in the 50–55 µm range, with two peaks around 71–75 µm and 76–80 µm. As for SMPs, the smallest size reached 5 µm (Figure S7).

Some components exhibit specific temporal patterns, in which certain compounds are consistently present throughout all months (such as some fillers and pigments), while others appear to be associated with seasonal variability, occurring only during specific periods of the year. For instance, fatty acids, alcohol esters, and glycerol monostearate (with a few exceptions) were detected almost exclusively during the cold months and in relatively high concentrations, suggesting a seasonal source or usage pattern.

### 2.3. Non-Targeted Analysis of PAs by HPLC-HRMS

HPLC-HRMS non-targeted screening (NTS) was applied to a selected winter window (December 2023–February 2024) as a proof-of-concept to demonstrate the analytical integration of particulate and dissolved additive profiling. Consequently, HRMS data are intended to illustrate phase distribution and chemical diversity rather than to provide an annual seasonal assessment of plastic additives.

NTS revealed a pronounced disparity between fractions: the dissolved phase exhibited greater chemical diversity and overall peak area compared to the particulate phase. While the methanol extraction protocol was selected to target polar to medium compounds from the solid fraction, additives were virtually absent in the particulate extracts. This suggests that the detected polar additives reside predominantly in the aqueous phase (Figure 3). Conversely, the presence of non-polar organic species associated with the particulate matter, which are less efficiently recovered by methanol extraction, was qualitatively supported by Micro-FTIR analyses.

In the samples, different types of chemicals were detected, including pharmaceuticals, personal care products, biocides, biomolecules, drugs of abuse and their metabolites, and, of course, plastic additives, which are the focus of this study (Supplementary Information, SI).

Many identified chemicals serve multiple purposes across sectors; for instance, dibutyl phosphate is used as a plasticizer, antistatic agent, dielectric, antifoaming agent, and hydraulic fluid [54,55].

Compounds were selected based on their fragmentation, signal intensity and reliability of the spectral match with selected databases (Table S3). A selection of matching spectra is shown in Figures S8–S10. The identified PAs are reported in Table 1.

Most of the PAs identified in the samples are plasticizers and flame retardants, which are among the most widely produced and sold additives worldwide [12,14]. Some of the compounds that could be identified at level 1 or 2a [56] were Organophosphorus Esters (OPEs), such as Tributyl Phosphate (TBP), Triphenyl Phosphate (TPP), and Tris(2-chloroethyl) Phosphate (TCEP), widely used as flame retardants and plasticizers [57,58,59]. Besides these compounds, other OPEs were present in all the samples but could not be identified. However, many spectra showed the presence of the diagnostic fragment at 98.9845 *m*/*z* of [H_4_PO_4_]^+^ [60].

Other plasticizers that could be identified at level 1 or 2 are N-Butylbenzenesulfonamide (NBBS), diethyl phthalate (DEP), and acetyl tributyl citrate (ATBC), also known as Citroflex A-4.

NBBS was found in the sample of February. This sulfonamide serves as a versatile plasticizer in the production of various polymers, including polycarbonate, polyamine, melamine, urea, copolyester and phenolic polymers (Wypych, 2017 as cited in Johnson et al., 2023 [61]). NBBS exhibits multi-system toxicity (neuro, immune, and general): initially identified as a neurotoxin due to plasticware leaching [62], subsequent studies characterized its rapid absorption, brain penetration [63], and immunomodulatory effects at high doses. However, toxicological effects, including oral LD50 values of 1725–3560 mg/kg [61], consistently occur at exposure levels orders of magnitude higher than typical environmental concentrations (ng/L–μg/L range).

DEP, detected in the December and February samples, is mainly used as a plasticizer in polyvinyl chloride (PVC) [64,65] and is found in a wide range of plastic products, including toys, toothbrushes, plastic films, and automotive components [66]. It is also used as a solvent and as a carrier for fragrances in personal care products [67]. Phthalates are considered endocrine-disrupting chemicals; due to their structure, they can mimic hormones and cause various abnormalities, such as precocious puberty and endometriosis. DEP, in particular, has been shown could induce developmental, reproductive, and liver toxicity in male rats, while there is limited evidence of reproductive toxicity in female rats [66].

ATBC is a non-toxic, medical-grade plasticizer [68]. Also, due to its low migration rate [69], it is among the substances used to replace phthalates in PVC, including for medical devices and food-contact packaging [70].

Hexamethoxymethyl melamine (HMMM) was detected in January and February samples. HMMM is a cross-linking agent used in the synthesis of thermotolerant coatings, plastics and tires [71], commonly found in cans, coils, vehicle components, fences and various objects in urban areas [72,73,74]. Although frequently detected in TWP leachate, its European release stems mainly from plastics, coatings, and industrial wastewater [75]. While HMMM exhibits low acute aquatic toxicity, it is not readily biodegradable and has shown genotoxic potential in mammalian cells in vitro [73].

The majority of these compounds are degradable. DEP is mainly degraded by microorganisms, and the process appears to be faster under aerobic conditions [76,77,78]. HMMM is also biodegradable, although its degradation mainly occurs in wastewater treatment plants. The degradation rate strongly depends on sludge pH, with reported half-lives ranging from 3.3 h under acidic conditions to approximately one year at higher pH values [73]. ATBC is likewise biodegradable [79], whereas NBBS is persistent [61].

Benzotriazole, N,N-diphenylguanidine, hydroxybenzothiazole, and hydroxybenzophenone are also used as additives in the plastic industry and were detected in the samples. Identification included level 2 for benzotriazole and N,N-diphenylguanidine, while the other spectra were assignable to the hydroxybenzothiazole and hydroxybenzophenone congeners.

Other PAs were also detected, despite falling below strict signal intensity filtering criteria. Laurolactam (a polyamide monomer) was tentatively identified in February, while uncharacterized fatty acid esters were observed across all samples. Bisphenol B, not manufactured in Europe but present in imported goods [80,81], was identified in December. Additionally, polyethylene glycol (PEG) chains of varying lengths were present; widely used in pharmaceuticals and cosmetics, PEGs are generally considered safe due to low toxicity and biocompatibility [82]. Specifically, PEG n7 was confirmed via the mzCloud database (confidence level 2a), whereas shorter PEGs were tentatively identified through characteristic fragment peaks at *m*/*z* 89.06, 133.09, and 177.11 [83,84].

While these toxicity and biodegradability profiles provide valuable context regarding the detected chemical classes, they are included for descriptive purposes only, as quantitative target analysis and ecotoxicological assays were beyond the scope of the present study.

## 3. Discussion

### 3.1. Microplastic Abundance and Distribution: Comparison with Literature

Our results on MP characterization and counting align with recent literature showing that MPs < 100 µm dominate environmental matrices [85,86,87]. Similarly, MPs in China’s Taihu Lake Basin were mainly 20–200 µm, with 20–100 µm fibers predominant [88], while Seine River particles averaged 53 µm (minor) and 96 µm (major) [89].

This prevalence of fine-sized MPs raises concerns regarding transport, bioavailability, and uptake by small organisms [90]. However, direct comparisons remain difficult due to non-standardized methodologies—including variations in sampling, extraction, detection limits, and reporting units [91]. This fragmentation particularly affects smaller fractions (<10 μm), which remain underrepresented despite being highly toxic and abundant [92]. In terms of polymer composition, PE and PP consistently dominate freshwater ecosystems due to high global production [91]. Reported abundances vary widely depending on mesh size and method, ranging from 0.001–200 MPs/L to 12,000 MPs/m^3^ [93,94], or up to 1556 MPs/L in lakes [95]. Similar variability occurs in drinking water, with European tap water spanning 0.0007–628 MPs/L [96] and Lisbon averaging 309 MPs/L [97] targeting sub-micron particles (<1 μm) shifts counts significantly higher—e.g., 1473–3605 particles/L in raw river water, dropping to 338–628 post-treatment [98]—with regional concentrations reaching 1756–5422 MPs/L in India [99].

To ensure comparability with existing literature, TWPs were excluded from our baseline analysis. Quantitative research on freshwater TWPs remains scarce [38] and often relies on mass units [100] rather than particle counts. By focusing on standard SMPs, our detected concentrations (1084–3794 particles/L) align with the higher range of studies targeting smaller size fractions [97,98]. Ultimately, these discrepancies demonstrate how size cut-offs, reporting units, and particle selection fundamentally bias quantification, underscoring the urgent need for a harmonized analytical framework.

### 3.2. Non-Targeted and Suspect Screening: Comparison with Literature

Also NTS and Suspect Screening (SS) results align with existing literature. SS in the Rhône River (France) confirmed the presence of DEP, TPP, and TCEP [101]. Similarly, analysis in the Besòs River (Spain) detected DEP—at lower concentrations than phenols and benzophenone—while ATBC was not detected [102]. Notably, both studies reported diethylhexyl phthalate as the most abundant compound, whereas it was absent in our samples. Furthermore, NTS across European rivers revealed numerous matching additives, including NBBS, TBP, TCEP, TPP, triethyl phosphate, and DBP [103]. Non-PA compounds, such as certain pesticides, were also consistently identified in our samples (Supplementary_Information S2).

### 3.3. SMPs, MLCs and PAs Results: A Complementary Analytical Approach to Plastic-Associated Additives

Beyond polymeric particles, solid-phase PAs represent an emerging, underexplored facet of microplastic research that requires an integrated multi-approach methodology. Micro-FTIR and HPLC-HRMS serve as complementary tools: Micro-FTIR identifies hydrophobic compounds embedded within the solid polymer matrix, while HPLC-HRMS, using polar extraction solvents, detects hydrophilic loads in the dissolved phase. Integrating these parallel methods enhances insights into PA migration, bioavailability, and physicochemical behavior, establishing a robust framework to correlate specific additives with their plastic matrices in future large-scale datasets.

This analytical distinction between particulate and dissolved phases carries relevant environmental implications for strategic water resources. Investigating a freshwater canal feeding a drinking water treatment plant revealed the concurrent presence of SMPs, MLCs, and PAs, highlighting a potentially underestimated exposure pathway for drinking water where current quality standards overlook both particles < 100 µm and their non-covalently bound additives.

Beyond their individual presence, the simultaneous occurrence of microplastics and chemical additives raises valid concerns regarding potential interactive and synergistic toxicity. Joint-exposure studies demonstrate that interactive risks in polymer–additive mixtures can substantially exceed individual hazard estimates, often governed by polymer type and environmental weathering [104]. For instance, while some polymers exert risk mainly through direct physical hazards, others exhibit strong interaction-driven toxicity when co-occurring with additives such as phthalates. Furthermore, environmental aging can exacerbate these combined impacts: surface oxidation and increased roughness of weathered microplastics not only enhance physical tissue damage but also facilitate the sorption, accumulation, and leaching of co-pollutants (e.g., flame retardants), leading to aggravated oxidative stress and metabolic disruption in organisms [105]. Even when specific additives evaluated in laboratory models are absent, the co-existence of fine microplastics and detected additives (e.g., organophosphates, phthalates) in the analyzed system suggests a potential for synergistic adverse effects on aquatic biota.

Consequently, these findings provide valuable insights for future regulatory directives and monitoring protocols. Integrating emerging pollutants into freshwater risk assessments and establishing standards for synergistic contaminant mixtures is necessary, in line with EU Directive 2020/2184 [34]. While Commission Delegated Decision (EU) 2024/1441 [35] standardized measurement methodologies—acknowledging MP concentrations up to 400 particles/L in drinking water—the current framework remains limited to monitoring. Future regulatory frameworks could systematically consider transitioning from observation to regulation by exploring science-based concentration limits. Such thresholds must address both physical particle toxicity and chemical hazards, recognizing microplastics as dynamic vectors capable of leaching harmful additives into the human body.

## 4. Materials and Methods

### 4.1. Sampling Site and Collection Campaign

The sampling campaign was carried out in the framework of the project “BSL6- Drainage Basin of the Venice Lagoon pursuant to the special regulations for Venice and Directive 2000/60/EC [106], and monitoring of microplastics in the Drainage Basin and the Venice Lagoon”. Water was collected monthly from March 2023 to March 2024 at the intake of the Ca’ Solaro drinking water treatment plant (Venice), fed by the 10.5 km open-air CUAI canal diverting water from the Sile River across agricultural and urban areas (Figure 4). Using a custom-designed, pre-decontaminated glass bottle sampler [4], 7.5 L of water were collected by lowering the device at 15 min intervals. Samples were stored in pre-decontaminated 2.5 L amber glass bottles at 4 °C until laboratory arrival. The contents of the three 2.5 L bottles were pooled to obtain a single sample representative of each month. Of the resulting 7.5 L sample, 5 L were allocated to SMP and MLC analysis, from which three 1 L laboratory replicates were prepared and filtered separately (1 L per filter). The remaining 2.5 L were allocated to the non-targeted and suspect analysis.

### 4.2. Pre-Treatment of Water Samples for Micro-FTIR Analysis

Detailed Quality Assurance and Quality Control (QA/QC) protocols are provided in the Supplementary Information (SI). Briefly, strict contamination-minimization measures were adopted throughout the workflow by utilizing glass and stainless-steel equipment and avoiding plastic materials. Procedural and reagent blanks analyzed alongside the samples showed no synthetic particles or fibers, confirming a controlled background. Furthermore, spike-and-recovery tests yielded an optimal average recovery rate (>90%, i.e., 95%). ensuring that particle losses during pretreatment were minimal.

SMPs and MLCs in the water samples were extracted through the oleo-extraction procedure previously developed by Corami et al., 2021 [46], with slight modifications (SI).

A glass vacuum filtration system and aluminum oxide filters (0.2 μm, 47 mm diameter, further details in SI) were employed for the filtration of all oleo-extracts. According to Corami et al., 2020 [46,107], the purification procedure was performed during the filtration. Each filter was stored in decontaminated glass Petri dishes slightly open and left to dry for at least 72 h under a decontaminated laminar flow hood. Then, the filters were carried to the instrumental lab.

### 4.3. Quantification and Chemical Identification of SMPs and MLCs via Micro-FTIR

The simultaneous analysis of SMPs and MLCs was carried out using a Micro-FTIR Nicolet™ iN™ 10 (Thermo Fisher Scientific, Waltham, MA, USA), equipped with an ultra-fast motorized stage and liquid nitrogen-cooled mercury cadmium telluride detector (MCT). Using the PARTICLES WIZARD section of the Omnic™ Picta™ software (v 1.8.259), Particles Analysis, which allows the simultaneous quantification and identification of SMPs and MLCs, was performed (SI) [46,107,108]. Particle size in micrometers was determined automatically using the Particles Wizard module of OMNIC Picta software, defined by the smallest bounding box enclosing the particle, from which the Aspect Ratio was calculated to classify particle morphology (sphere, ellipse, or cylinder) (further details in Supplementary Information). For the identification of TWPs, microFTIR spectra were matched against a dedicated reference library built using authentic reference materials obtained from known tyre treads and generated TWPs [44,104]. A minimum spectral match threshold of 70% was set. To prevent misidentifications from asphalt binder fragments or general rubber-containing debris, morphological features (such as mineral encrustations) were assessed. Furthermore, this micro-FTIR screening approach was previously cross-validated and benchmarked against ATR-FTIR, Pyrolysis-GC-MS and SEM-EDS analyses, ensuring high selectivity for TWP quantification as detailed in [44]; for more information see SI.

### 4.4. HPLC-HRMS Analysis of PAs

To complement Micro-FTIR analysis for dissolved-phase compounds, PAs were analyzed by mass spectrometry on selected samples (December 2023, January and February 2024). Samples stored at –20 °C were thawed, and 1 L per sample was filtered through 1.2 µm glass fiber filters (GFF, 47 mm, Whatman, UK; ~500 mL per filter). Filters were fragmented and extracted in glass vials using MeOH (5 mL, 2 mL, and 4 mL) with 15 min ultrasonication per step. Combined extracts (~11 mL) were filtered (0.22 µm cellulose acetate, Ø 25 mm), evaporated to 1 mL under N_2_ (1.2 bar, 45 °C; Turbovap II^®^, Caliper Life Science, Hopkinton, MA, USA), and diluted 1:1 with ultra-pure water (UW). For the filtered aqueous phase (1 L), solid-phase extraction (SPE) was performed using Oasis HLB cartridges (200 mg, Waters, Etten-Leur, Netherlands) conditioned with 5 mL MeOH and 5 mL UW, eluted with 5 mL MeOH, and diluted 1:1 with UW.

Samples were analyzed via HPLC-HRMS using a Vanquish system coupled to an Orbitrap Exploris 120 mass spectrometer (Thermo Fisher Scientific, Waltham, MA, USA). Separation was achieved on a Zorbax SB-Aq C_18_ column (2.1 × 150 mm, 3.5 µm; Agilent, Santa Clara, CA, USA) at 200 µL/min with mobile phases A (0.01% formic acid in UW) and B (0.01% formic acid in acetonitrile). The 80 min gradient (B%) was: 0–5 min 0%, 45–60 min 100%, and 63–80 min 0%.

The mass spectrometer operated in positive (3500 V) and negative (2500 V) H-ESI modes (sheath gas: 25 Arb; aux gas: 5 Arb; transfer tube: 320 °C; vaporizer: 300 °C). Full-scan HRMS data (*m*/*z* 85–1000, resolution 120,000 at *m*/*z* 200) were acquired using data-dependent acquisition (DDA) with stepped HCD collision energies (30, 70, 90).

Data were processed with Compound Discoverer (Thermo Fisher Scientific, Waltham, MA, USA) following the workflow in Figure S4 and prioritized using filtering criteria (Table S3). Spectra were manually verified against the mzCloud database and an in-house internal standard database, with identification levels assigned per Schymanski et al., 2014, criteria [56].

### 4.5. Statistical Analyses

Data on SMP and MLC abundances follow the Poisson distribution [56,109] and the confidence interval (fiducial interval, FI, which is the measurement uncertainty (measurement error)) was calculated accordingly. The F test (α = 0.05) was performed to test whether variances were homogeneous. The Kruskal–Wallis test (*p* < 0.05), a non-parametric statistical test, was performed to assess the meaningfulness of the differences observed, having verified that the variances were not homoscedastic. Statistical analyses were performed using STATISTICA software (v 14.3) (TIBCO, Palo Alto, CA, USA).

## 5. Conclusions

This study highlights the value of a complementary, multi-analytical approach to characterize plastic pollution and associated additives in freshwater systems. Monthly monitoring of an artificial canal feeding the Ca’ Solaro drinking water treatment plant in Venice confirmed the year-round presence of microplastics (including TWPs), peaking in autumn and winter, with PTFE, HDPE, and PA6 emerging as the dominant polymers. Importantly, combining Micro-FTIR and HPLC-HRMS revealed a distinct phase distribution of PAs: the particulate phase was dominated by lubricants, fillers, and antistatic agents, whereas the dissolved phase exhibited a high diversity of polar PAs, primarily plasticizers and flame retardants.

These findings point to a potential exposure pathway to sub-100 µm plastics, MLCs, and PAs that is currently not fully captured by routine water monitoring standards. To translate these insights into broader applications, key challenges should be addressed, including expanding spatial and temporal sampling to account for environmental variability and tracking the purification process through the treatment plant to assess effluent removal efficiency. Future research would also benefit from investigating the chemical degradation and weathering of these complex additive blends. Ultimately, this study emphasizes the utility of integrated monitoring frameworks that treat particulate and dissolved contaminants as an interconnected system, providing valuable support for improving filtration strategies and safeguarding drinking water resources.

## Figures and Tables

**Figure 1:**
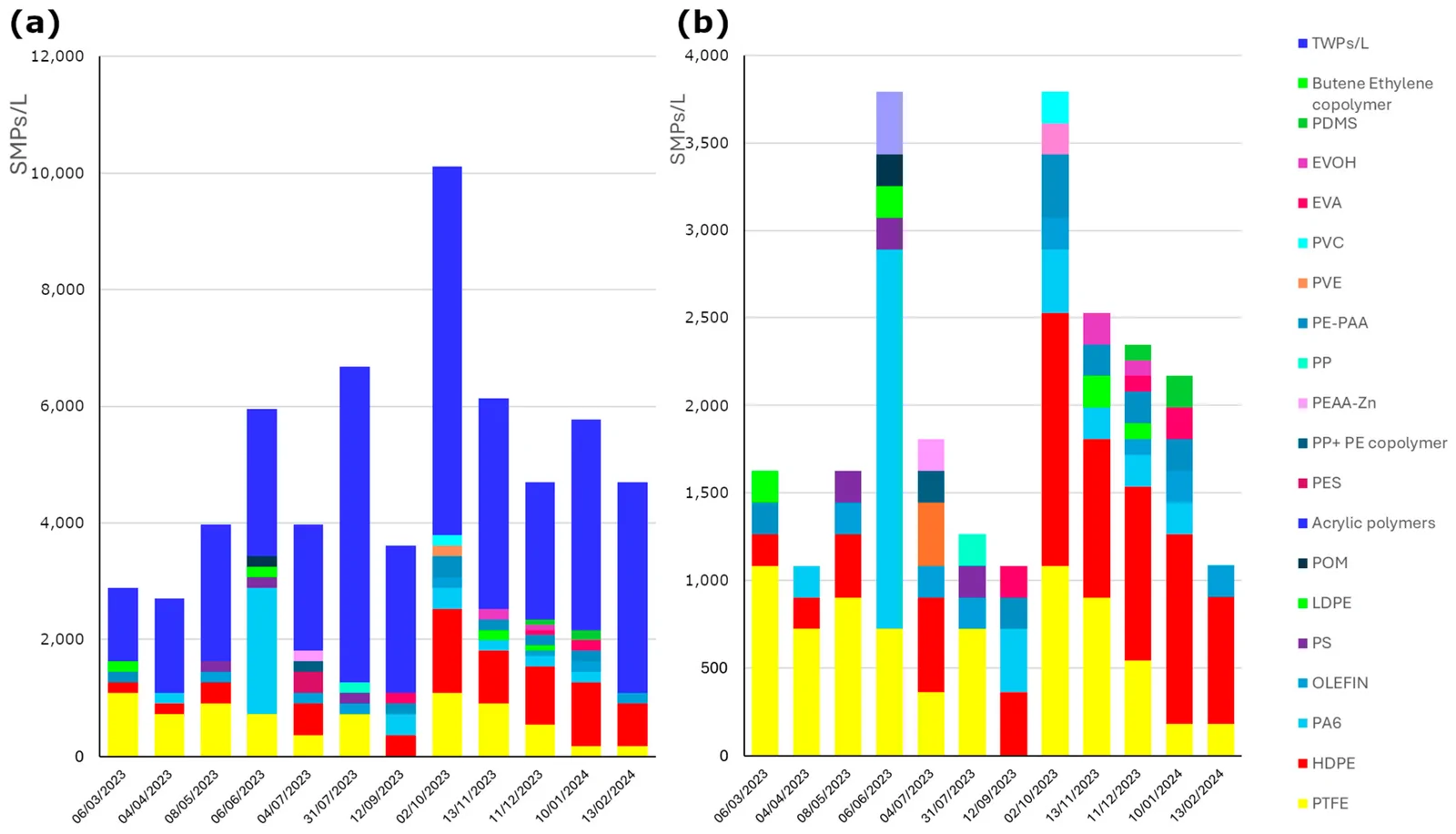
Abundance of SMPs (particles/L) detected in the samples. (**a**) Total abundance of all SMPs, including tire wear particles (TWPs). (**b**) Abundance of the remaining polymer types after excluding TWPs, in order to improve the visualization of the other polymer category.

**Figure 2:**
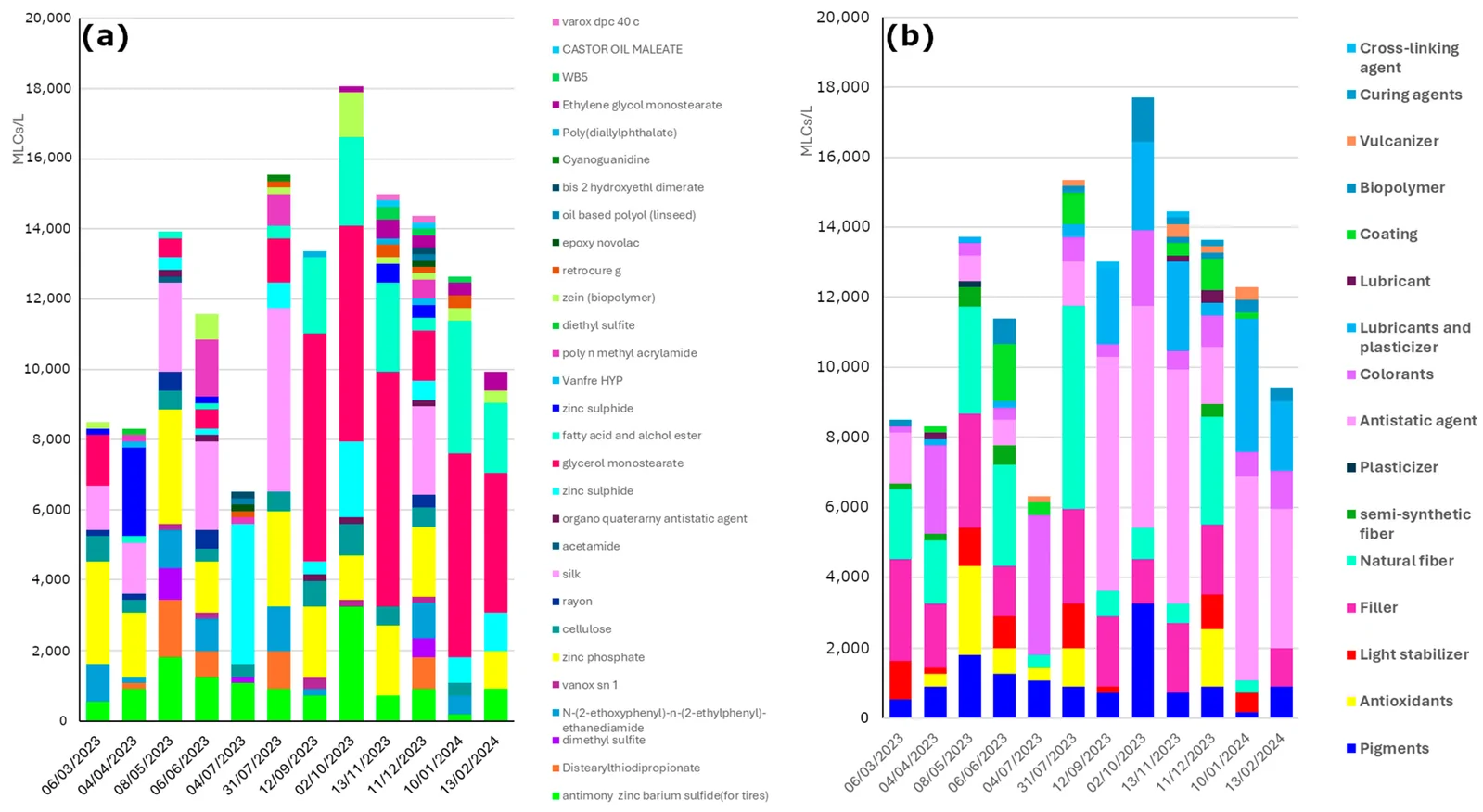
Abundance of MLCs (MLCs L^−1^) detected in the samples. (**a**) Abundance of individual MLC compounds identified through Micro-FTIR analysis. (**b**) Abundance grouped by MLC categories.

**Figure 3:**
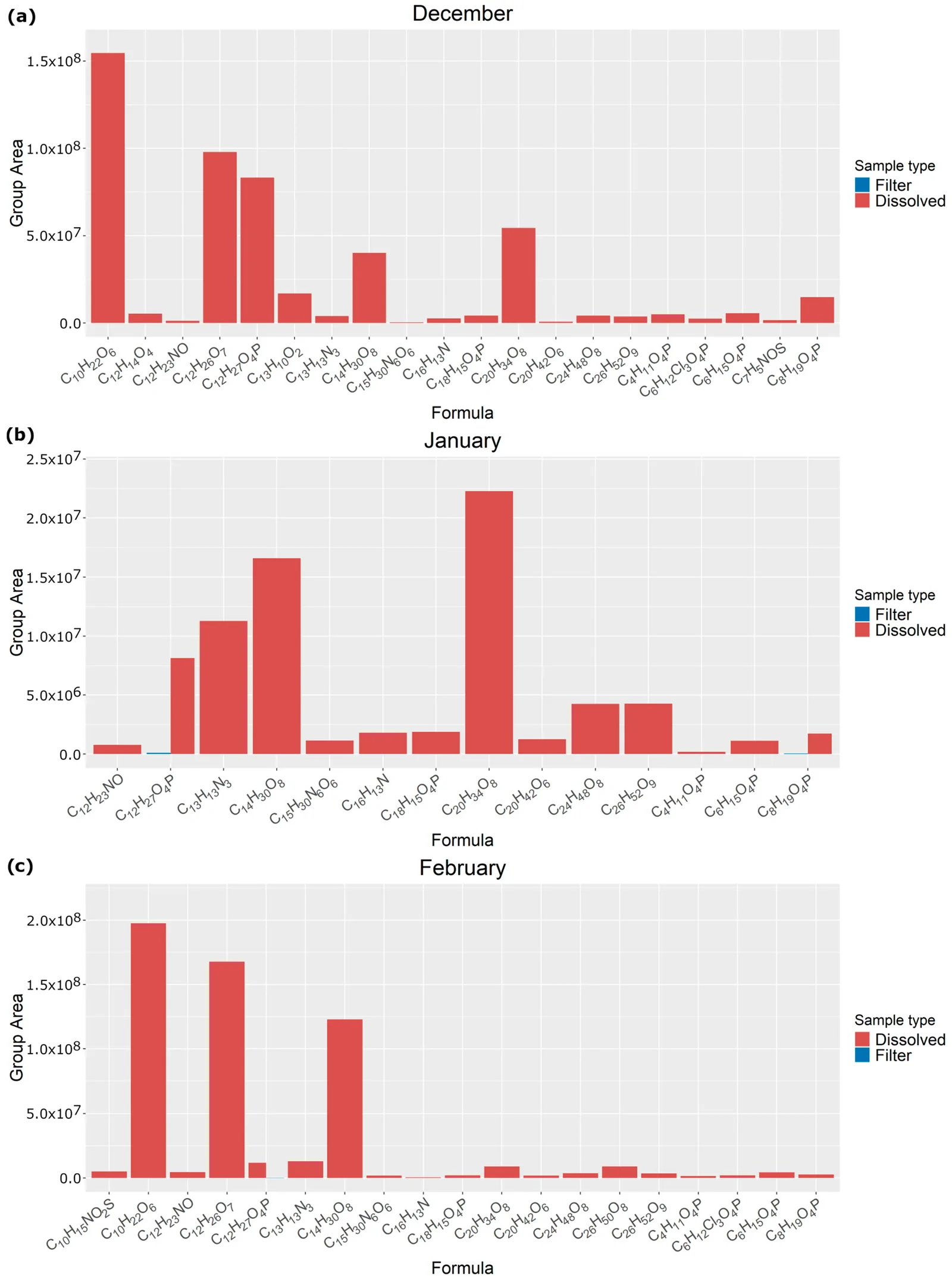
Comparison of detected compound abundances (expressed as Group Area) between the dissolved phase (red) and the filter phase (blue) during (**a**) December, (**b**) January, and (**c**) February. The signal is heavily dominated by the dissolved fraction, with the filter phase showing negligible to non-detectable levels across all identified chemical formulas.

**Figure 4:**
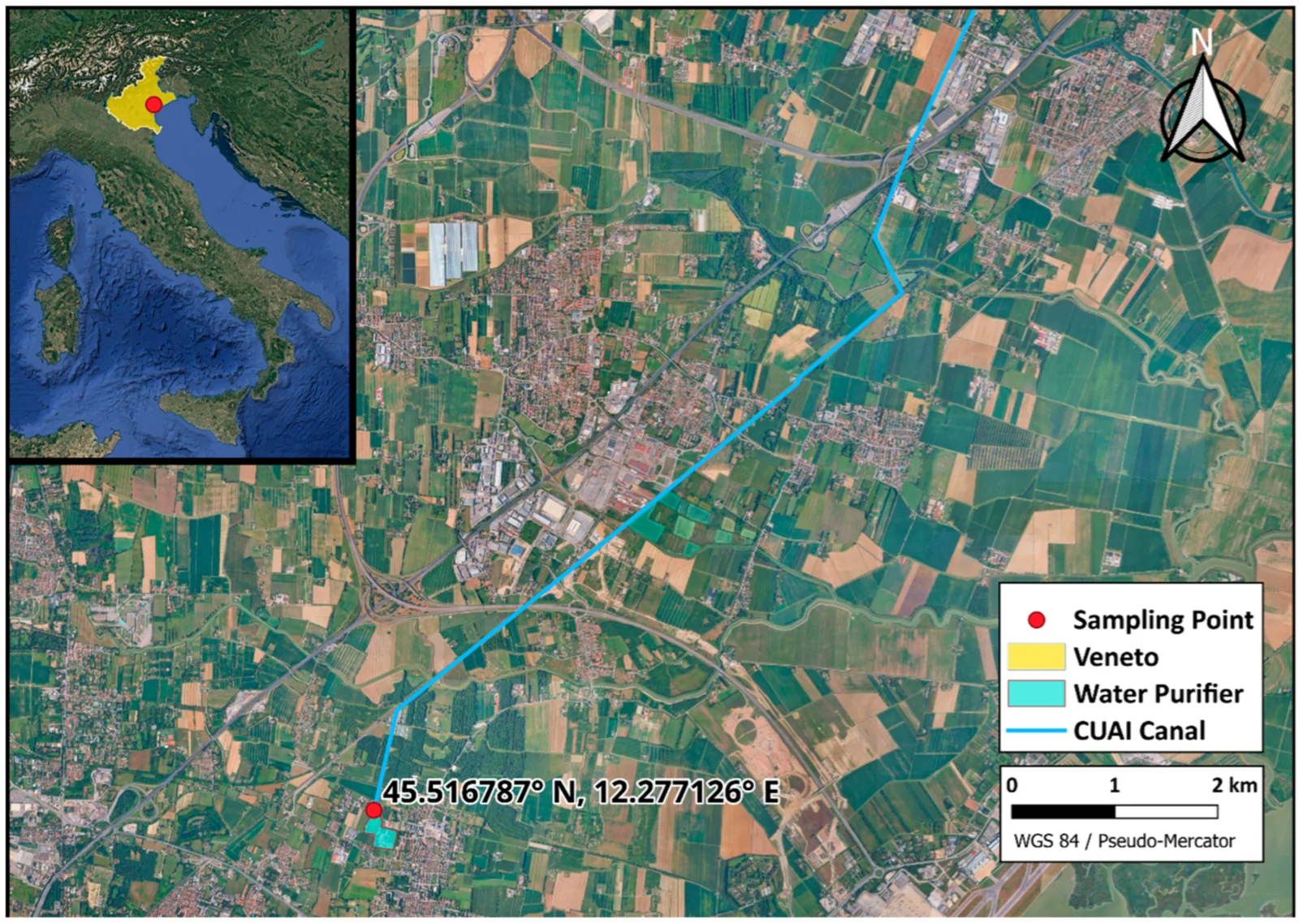
Geographic location of the study area and the sampling point (red dot; coordinates: 45.516787, 12.277126) located near the water purifier in the Veneto region, Italy. The blue line represents the CUAI Canal. The inset map (top-left) shows the regional context within Italy.

**Table 1:** Identified and tentative candidates of PAs and relative presence in the samples.

Molecule	Calculated Molecular Weight	ID Level	Formula	December	January	February
**2-Hydroxybenzothiazole**	151.009	1	C_7_H_5_NOS	X		
**Diethyl phthalate**	222.0889	1	C_12_H_14_O_4_	X		X
**Triphenyl phosphate**	326.0702	1	C_18_H_15_O_4_P		X	X
**Dibutyl phosphate**	210.1017	2a	C_8_H_19_O_4_P	X		X
**N,N’-Diphenylguanidine**	211.1107	2a	C_13_H_13_N_3_	X	X	X
**N-Phenyl-2-naphthylamine**	219.1044	2a	C_16_H_13_N		X	
**N-Octyl-2-pyrrolidone**	197.1777	2a	C_12_H_23_NO	X		X
**Tributyl phosphate**	266.1642	2a	C_12_H_27_O_4_P	X	X	X
**Tris**(**2-chloroethyl**) **phosphate**	283.9534	2a	C_6_H_12_Cl_3_O_4_P	X		X
**4-Hydroxybenzophenone**	198.0678	2a	C_13_H_10_O_2_	X		
**PEG n7**	326.1934	2a	C_14_H_30_O_8_	X	X	X
**Hexamethoxymethyl melamine**	390.222	2a	C_15_H_30_N_6_O_6_		X	X
**Citroflex A-4**	402.2245	2a	C_20_H_34_O_8_		X	X
**N-Butylbenzenesulfonamide**	213.0821	2a	C_10_H_15_NO_2_S			X
**Triethyl phosphate**	182.0704	2a	C_6_H_15_O_4_P	X		X
**PEG**	282.1673	3	C_12_H_26_O_7_	X		X
**Organophosphate**	154.0393	3	C_4_H_11_O_4_P	X		
**PEG**	238.1412	3	C_10_H_22_O6	X		X
**Polyether**	378.2975	3	C_20_H_42_O6		X	
**Organophosphate**	182.0705	3	C_6_H_15_O_4_P		X	
**Organophosphate**	210.1018	3	C_8_H_19_O_4_P		X	
**Organophosphate**	210.1017	3	C_8_H_19_O_4_P		X	
**PPG**	308.2194	3	C_15_H_32_O_6_			X
**Organophosphate**	154.0392	3	C_4_H_11_O_4_P	X		X
**PEG**	238.1414	3	C_10_H_22_O_6_			X

## Data Availability

Data will be made available on request.

## Supplementary Materials

The following supporting information can be downloaded at: https://www.mdpi.com/article/10.3390/molecules31213973/s1, Supporting Information S1: Additional experimental details, materials and methods, including explanatory figures (DOC); Supporting Information S2: Additional information on identified and tentatively identified features (xlsx). References cited in SI: [44,46,107,108,110,111,112,113,114,115,116,117,118,119,120,121,122,123,124,125,126,127].

## Abbreviations

The following abbreviations are used in this manuscript:

ATBC	Acetyl Tributyl Citrate
DBP	Dibutyl Phosphate
DDA	Data-Dependent Acquisition
DEP	Diethyl Phthalate
ECHA	European Chemicals Agency
EU	European Union
EVA	Ethylene-Vinyl Acetate
FTIR/Micro-FTIR	(Micro) Fourier-Transform Infrared spectroscopy
GFF	Glass Fiber Filter
HCD	High-energy Collision Dissociation
HDPE	High-Density Polyethylene
H-ESI	Heated Electrospray Ionization
HLB	Hydrophilic-Lipophilic Balance
HMMM	Hexamethoxymethyl melamine
HPLC	High-Performance Liquid Chromatography
HRMS	High-Resolution Mass Spectrometry
LDPE	Low-Density Polyethylene
*m*/*z*	Mass-to-charge ratio
MCT	Mercury Cadmium Telluride
MeOH	Methanol
MLC/MLCs	Microlitter Component/Components
MP/MPs	Microplastic/Microplastics
NBBS	N-Butylbenzenesulfonamide
NTS	Non-Targeted Screening
OPE	Organophosphorus Esters
PA/Pas	Plastic Additive/Plastic Additives
PA6	Polyamide 6
PE	Polyethylene
PEAA-Zn	Poly(ethylene-co-acrylic acid) zinc salt
PEG	Polyethylenglycol
PES	Polyester
PP	Polypropylene
PTFE	Polytetrafluoroethylene
PVC	Polyvinyl Chloride
QA/QC	Quality Assurance and Quality Control
SI	Supplementary Information
SMP/SMPs	Small Microplastic/Small Microplastics
SPE	Solid-Phase Extraction
SSA	Suspect Screening Analysis
TBP	Tributyl Phosphate
TCEP	Tris(2-chloroethyl) Phosphate
TPP	Triphenyl Phospate
TWP/TWPs	Tire Wear Particle/Particles
UW	Ultrapure Water
