# Supplementary material for: Tracking Small Microplastics and Plastic Additives in Freshwater: An Integrated Analytical Approach
Source: Molecules. 2026 Nov 4;31(21):3973. doi: 10.3390/molecules31213973 (PMC13765060; doi:10.3390/molecules31213973)
Supplement: Supplementary file 1 [file molecules-31-03973-s001.zip › SupplementaryInformation_S1.pdf]

Supplementary Information

Characterization of Small Microplastics and Plastic Additives in Freshwater: A Dual-Phase Mult technical Approach using Micro-FTIR and HPLC-HRMS

Greta Palombella[1,2], Beatrice Rosso[3,1,2], Elisa Scalabrin[2,1], Roberta Zangrando[2,1], Andrea Gambaro[1,2], Fabiana Corami[2,1]

[1] Department of Environmental Sciences, Informatics and Statistics, Ca' Foscari University, via Torino 155, 30172, Venice, Italy

[2] Institute of Polar Sciences - National Research Council (CNR-ISP), via Torino 155, 30172, Venice, Italy

[3] National Interuniversity Consortium for Environmental Sciences (CINSA), via Torino 155, 30172, Venice, Italy

Summary

Reagents 2
Library employed for Micro-FTIR 2
Quality Assurance and Quality Control (QA/QC) 2
Method 3
Recovery Test 3
Particles Analysis using MicroFTIR and microscopic counting 4
Supplementary Information: Detailed Protocol for TWP Identification, Spectral Library Setup ... 8
Compound Discoverer Analysis Workflow 10
Compound Discoverer Filter 11
Microplastics Acronyms 12
Best Micro-FTIR Spectra 13
MLCs reported as classes of compounds 14
MPs size distribution 15
MLCs Size distribution 16
Collection of Best HRMS Spectrta 17
Matching with reference standards 21

Reagents

Ultrapure water (UW) is produced by a Purelab Chorus 1 fed by the Purelab Chorus 2+ (RO/EDI/UV; Elga Lab Water, Veolia, High Wycombe, UK).
Hydrogen peroxide (H_2O_2, 30%, ACS reagent), heptane (puriss. p.a., ACS reagent, reag. Ph. Eur., ≥ 99% (GC)), ethanol (absolute, for HPLC, ≥ 99.8%,), methanol (for HPLC ≥ 99.9%), and aluminum oxide filters (0.2 μm, 47 mm diameter, supported, ANODISC (Anopore Inorganic Membrane Whatman) were purchased from Merck (Darmstadt, Germany).
Citranox® acid detergent (Aldrich, Merck, Darmstadt, Germany) was employed to wash the glassware.
Organic solvent-free and cold-pressed sunflower seeds oil (SSO, Crudolio, Camisano Vicentino (VI) was employed for the oleoextraction.

Library employed for Micro-FTIR

Synthetic fibers by Microscope
Plastic Fibers
Polymer additives and plasticizers
HR Polymer additive and plasticizers
HR Hummel Polymer and plasticizers
HR Nicolet Sampler Library
HR Sprouse Polymer additives
HR rubber compounding material
Tire wear and road particles (implemented, Rosso et al., 2023)
Bioplastics (implemented, Afshar et al., 2025; Corami et al., 2025)

Quality Assurance and Quality Control (QA/QC)

Several specific steps were implemented to minimize contamination throughout the sampling, transport, pretreatment procedure, and during the analysis. Throughout the process (from sampling to the analysis), the operators wore cotton lab coats, cotton shoe covers and nitrile gloves. All operations were performed in a plastic-free ISO 7 Clean Room, which is entirely made of stainless steel (e.g., floor, ceiling, workbenches, chemical fume hoods, etc.) and has limited access. The air circulation system is controlled, and the air filtration system is plastic-free.

The glass sampler was decontaminated and wrapped in decontaminated aluminum foil for transport to the sampling site. Ropes made of synthetic fibers were avoided; a plastic-coated electrical wire was employed. The wire was carefully rinsed with ultrapure water (UW), decontaminated with ethanol prior to use, and wrapped in decontaminated aluminum foil until the sampling time. Water samples were carefully stored at 4°C in a cooler bag till the arrival at the lab, where they were stored in the fridge until the pretreatment and analysis.

Glassware used for sampling and pretreatment was carefully washed and rinsed with UW, then decontaminated with methanol, followed by a 70% (v/v) methanol-ethanol solution, and finally rinsed with ethanol. A decontaminated glass box was used to collect the field blank, which was eluted with UW, filtered, and purified.

To monitor and minimize background contamination, strict quality assurance and quality control (QA/QC) measures were implemented throughout the entire analytical workflow. Glassware and stainless-steel tools were used and plastic-based components were avoided. Procedural blanks (including field blanks, reagent blanks, and procedural extraction blanks) accompanied every batch of samples. Specifically, ultrapure water (UW) and chemical reagents used during digestion/extraction were processed identically to the samples. No synthetic particles or fibers were detected in any of the procedural or reagent blanks, confirming that background contamination from the laboratory environment and reagents was negligible and well-controlled. Consequently, no blank subtraction was required.

A recovery test was performed by spiking three further replicates of one of the samples under examination with silver-grey PA 12 (polyamide 12) particles in the same size range as SMPs in the samples (further details in the supplementary information) to be analyzed via Micro-FTIR. The average yield of the recovery test was optimal (>90%, i.e.,95%). The pretreatment method was therefore efficient, accurate, and replicable.

As for the HPLC-HRMS analyses, in order to ensure high accuracy of m/z measurements, full range mass calibration was performed before the sample batch and one point mass calibration, based on Fluoranthene, was carried out at each injection. To check for instrumental signal variations, control samples were analysed repeatedly during the sample sequence; each sample was analysed in three technical replicates. Procedural blanks were produced and analysed to evaluate the baseline signal and highlight the presence of contaminants deriving from the sample treatment procedure or instrumental analysis.

Method

SMPs and MLCs in the water samples were extracted through the oleo-extraction procedure developed by Corami et al., 2021 [46], with slight modifications. Briefly, the collected water samples were homogenized to obtain a composite average sample representative of the bulk. Then, known volumes of water samples were diluted 1:1 with UW and poured into a glass separatory funnel to reduce water hardness and interferents. Then, 10 ml of H2O2 and 5 ml of Sunflower seed oil (SSO) were poured (further details of reagents in supplementary information), and the funnels were vigorously mixed for 10 minutes to obtain an emulsion. After 24 hours, the oleo-extract was carefully recovered and transferred to a decontaminated Erlenmeyer flask. In the same separatory funnel, 5 mL of H2O2 and 5 mL of SSO were added, and the procedure was repeated. After 3 hours, the aqueous phase was discarded, and the oleo-extract was recovered in the same Erlenmeyer flask using heptane and ethanol (1:1). The oleo-extraction of each sample was performed in triplicate.

Recovery Test

In order to assess the method’s yield, purchased particles of PA 12 (silver grey coloured, average size 90 μm; Goodfellow Cambridge Limited, Huntigdon, UK) were employed as a reference since polyamides are present

in different environmental matrices, such as biota, sediments, water, and atmosphere [44, 46, 110, 111]. Fortified samples were produced by spiking already characterized samples with 3.5 mg of PA12. The fortified samples were oleoextracted as samples. The average yield of the recovery test was above 90% (95%); hence, the pretreatment was efficient, accurate, and reproducible.

Particles Analysis using MicroFTIR and microscopic counting

Optical microscopy using dye staining, and electron and fluorescence microscopy are the standard non-invasive techniques employed to quantify MPs. However, these techniques are incredibly inclined to bias, and the identification of polymers is not provided. After a microscope investigation, some particles can be further analyzed with Fourier transform infrared spectroscopy (FTIR), and larger particles or fibers (>500 μm) can be analyzed by attenuated total reflection - FTIR (ATR - FTIR) since overestimation/underestimation of MPs can occur. Micro-FTIR is a well-known non-destructive technique that combines infrared spectroscopy with optical microscopy; it allows more reliable quantification (via microscopic counting) of MPs >100 μm and SMPs without overestimation/underestimation.

When each particle is unambiguously and optimally identified and measured, they can be counted according to the criteria of microscopic counting.

Microscopic counting has been employed for bacteria, phytoplankton, pollen, spores, and microplastics as well [112-122].

Microscopic counting can be employed with any filter suitable for analysis using Micro-FTIR and Micro-Raman (e.g., aluminum oxide filters, silicon oxide filters, etc.).

A significant advantage of microscopic counting is that there is no doubt how many organisms or cells, or particles are present within valid computable limits and degrees of chance.

When filters are employed as a support for counting, the measurement of complete filters is very time-consuming [116, 123]. However, analyzed filter areas, i.e., counting areas or count fields, need to represent the entire filter to avoid issues regarding representativeness and reproducibility.

Since the loading of the filters cannot be known in advance, counting areas with different abundances should be considered to avoid issues regarding the accuracy of extrapolation of findings of microplastics, organisms, cells, or bacteria.

The different approaches in choosing representative measurement areas are shown in Fig. S3.

In our study, a random approach has been applied.

The total counts from each filter must be multiplied by appropriate microscope conversion (optical factor F) and volume or dilution factors to provide the absolute abundance.

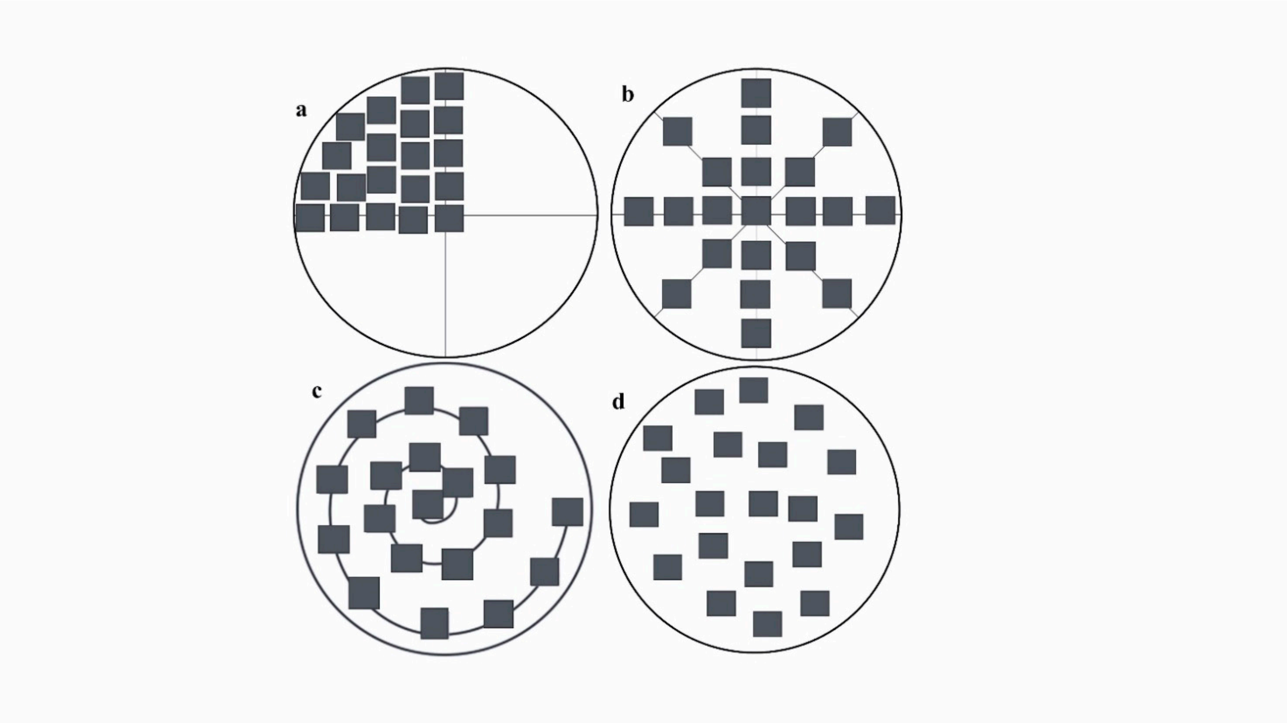

Figure S 1 (modified from Corami et al., 2021 [46]): the different ways in which microscopic counting can be performed, a) one sector, b) along the principal transects, c) from the center of the filter/slide/microscopic chamber towards the edges, d) random approach with no overlapping, which is the one employed in this study.

In the application note [108] it is fully reported how to perform Particles Analysis using the WIZARD section of Omnic™ Picta™.

Briefly, a count area (aka count field or mosaic) is collected using the Micro-FTIR microscope objective. Then, each count area is analyzed using the WIZARD section of Omnic™ Picta™, the instrument software. For each mosaic or count area selected, the image intensity histogram is essential to be able to select an adequate and significant number of particles since the amount of microplastics in that field is not known a priori; it detects the particles present on the surface of the filter in relation to the brightness value, i.e., the brightness ratio of each particle in contrast to the background (Figg. S2, S3).

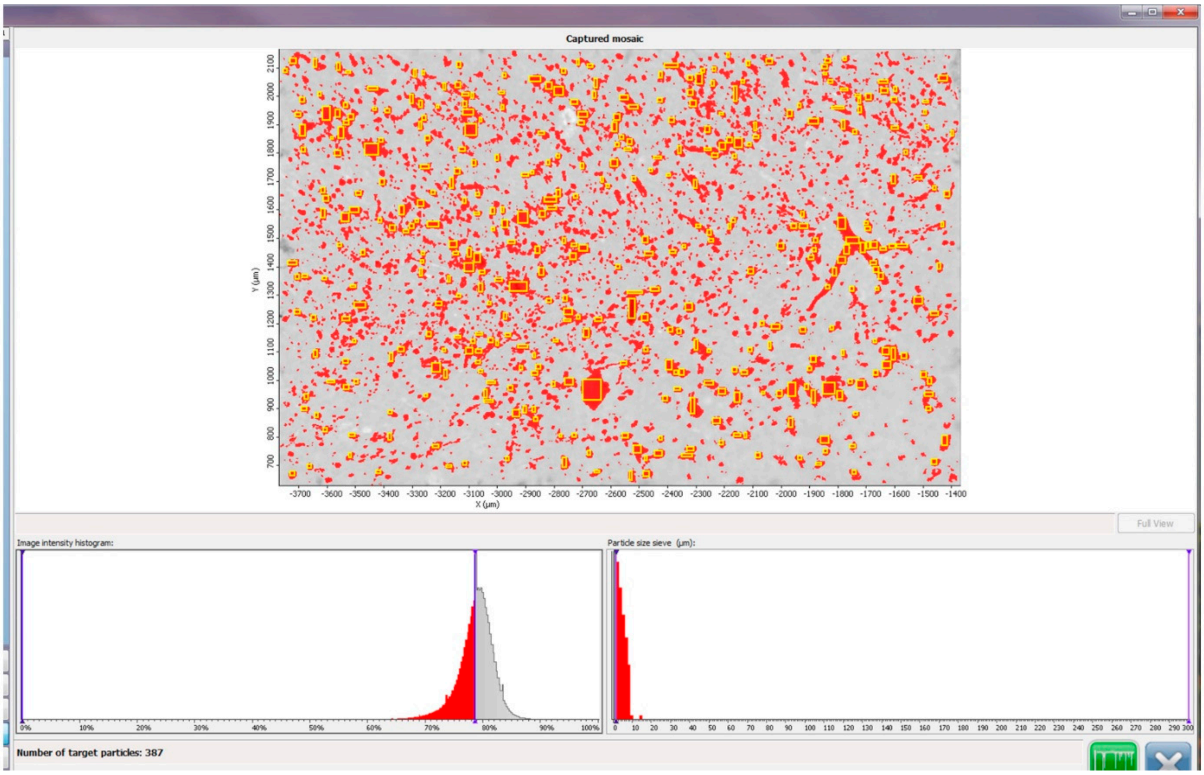

Figure S 2 - Example of particle selection on a count field for the Particles Analysis for simultaneous quantification and identification of particles. The image intensity histogram allows the selection of the particles according to their brightness.

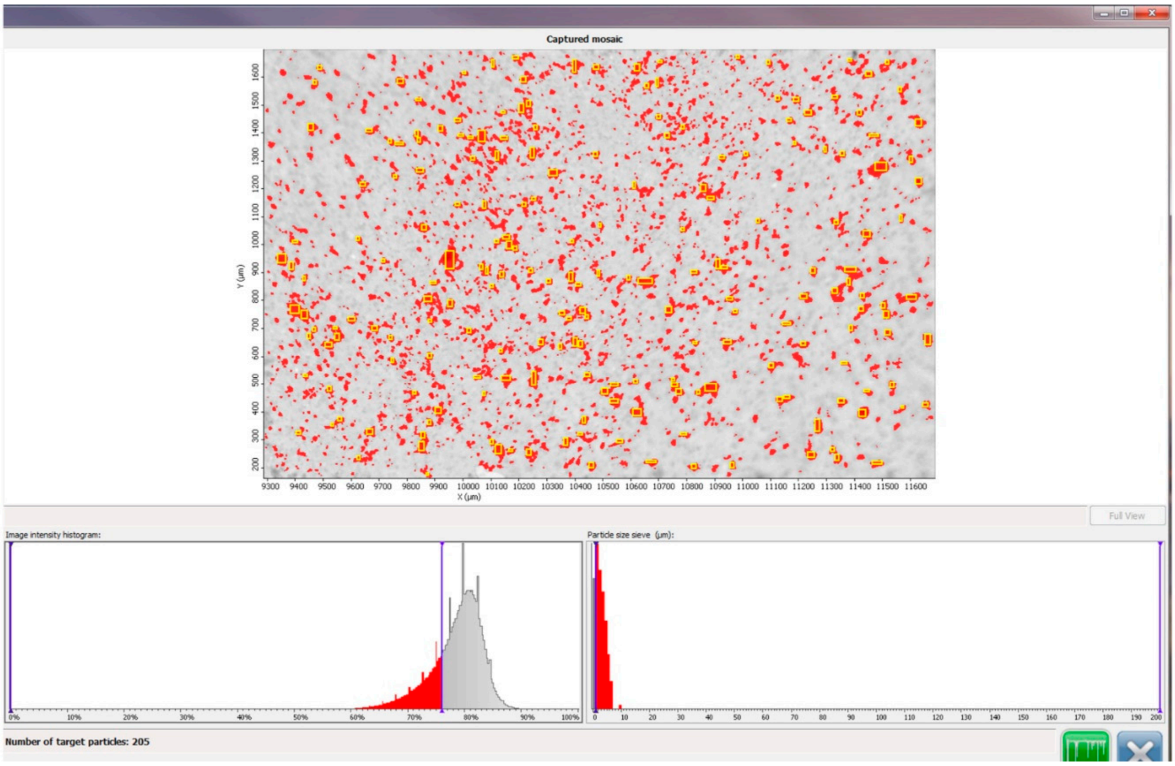

Figure S 3 - Another example of particle selection on another count field for the Particles Analysis for simultaneous quantification and identification of particles. The image intensity histogram allows the selection of the particles according to their brightness. Therefore, the number of particles selected can vary in different count fields.

Depending on the AR, particles, e.g., MPs and MLCs, can be approximated as regular geometric solids, e.g.,

spheres, ellipses, and cylinders, and their volumes can be calculated.

Since the particles have been unambiguously identified, the density can be retrieved. Therefore, the weight of the particles can be calculated, as well.

After the detection, raw spectra of particles are collected. After collecting these spectra, a background location is chosen for each count field, and the background spectrum is collected. By combining all the raw spectra with the background spectrum, the final result spectra are calculated and then identified by comparing them with spectra in the reference libraries. The match percentage indicates the similarity of each spectrum to each particle according to the similarity algorithm.

According to the instrumental characteristics, the optimal range of match percentage is ≥65%. The match percentage can reach 85% or even higher, thanks to oleoextraction and the purification procedure employed.

Each particle is enclosed in a bounding box, a polygonal frame (i.e., rectangle, square) that outlines its spatial extent. The bounding box is the smallest polygon enclosing the shape. The length and width of each particle are collected. The measured length and width will allow you to calculate the aspect ratio (AR) [107], a shape descriptor for the particles.

As a matter of fact, AR is defined as is the ratio between the maximum length (L) and the maximum width (W) of the smallest rectangle (bounding box) enclosing the shape:

$$AR = \frac{L_{max}}{W_{max}} \quad (S1)$$

When AR =1, particles are considered spherical, while when AR =2, particles are elongated/ellipsoidal, and they are considered elliptical. When AR ≥ 3 particles are considered cylindrical. Volumes of particles and fibers are then calculated according to their geometrical shape (i.e., sphere, ellipse, and cylinder).
Density, size, and shape are essential characteristics of particles and fibers that affect their transport and persistence in the environment, as well as their ingestion and/or inhalation. As observed for aerosol particles, microplastic particles and fibers can have different irregular shapes, which shape descriptors can then describe [124-127].

Briefly, quantification was performed via microscopic counting; a significant and robust number (at least 20) of known-sized areas (i.e., count fields, 2000 μm × 1200 μm) were randomly chosen, with no overlapping (Fig. S1). For each field, a significant and robust number of particles was selected in order to analyze a minimum total of 4,000 particles (further details in the supplementary information, Fig. S2). For each particle, the spectrum is retrieved, compared with spectra in several reference spectral libraries, and identified using a similarity algorithm (match percentage, match %; further details in the supplementary information). Furthermore, the length and width of each particle were measured, with a size LOD of 5 μm. Spectral identification was considered optimal when the match % was ≥65%; only particles optimally identified were quantified. Thanks to oleo-extraction, the particles often have a match percentage exceeding 70% (in some cases over 90%, Fig. S3); particles with a match percentage below 70% are double-checked (point-and-shoot analysis) to cross-validate the identification and quantification. The chemical identification of tire wear and road particles (TWRPs) was performed by comparison with the implemented reference library, which was obtained by the multi-analytical validation protocol previously developed [44]. Briefly, four different analytical techniques (ATR-FTIR, Micro-FTIR, Pyr/GC_MS and SEM) were employed to analyze a homemade standard and real environmental samples to provide a robust and unambiguous identification of TWRPs in complex environmental matrices.

Supplementary Information: Detailed Protocol for TWP Identification, Spectral Library Setup

Building upon the multi-analytical workflow previously established and validated by Rosso et al. (2023) [46], the identification and quantification of TWRPs in water samples were conducted under strict quality assurance and control criteria.

The microFTIR reference library was constructed using authentic reference materials, from tyre tread shavings collected from a range of commercially available vehicle end-of-life tyres (passenger cars and light commercial vehicles). During automated scanning via the Omnic™ Picta™ software (Particles Wizard module), a strict minimum spectral match threshold of 70%was enforced. Any particle yielding a match score below this threshold was automatically flagged for manual spectral inspection or excluded as unassigned debris. Due to the complex, heavily weathered, and carbon black-rich nature of TWPs, a combination of chemical and morphological criteria was applied to ensure high selectivity. Real environmental water samples contain complex mixtures of organic matter, minerals, and debris that can severely mask or alter the spectral response of rubber particles. To ensure correct identification, real samples underwent a rigorous pretreatment workflow [44], which successfully digested organic interferences and separated dense mineral fractions. This crucial step isolated the rubber-based micro-particles, allowing them to be correctly identified without matrix

interferences. Then, to guarantee the robustness of the microFTIR method, the screening protocol was previously benchmarked and cross-validated against independent analytical techniques [44]: Pyrolysis-Gas Chromatography-Mass Spectrometry (Py-GC-MS): Used for the targeted detection of characteristic thermal degradation markers (e.g., specific diene dimers/trimers and vulcanization byproducts) to confirm the polymeric composition of selected subsets; Scanning Electron Microscopy with Energy Dispersive X-Ray Spectroscopy (SEM-EDS): Employed to analyze surface morphology (shape and size).

Compound Discoverer Analysis Workflow

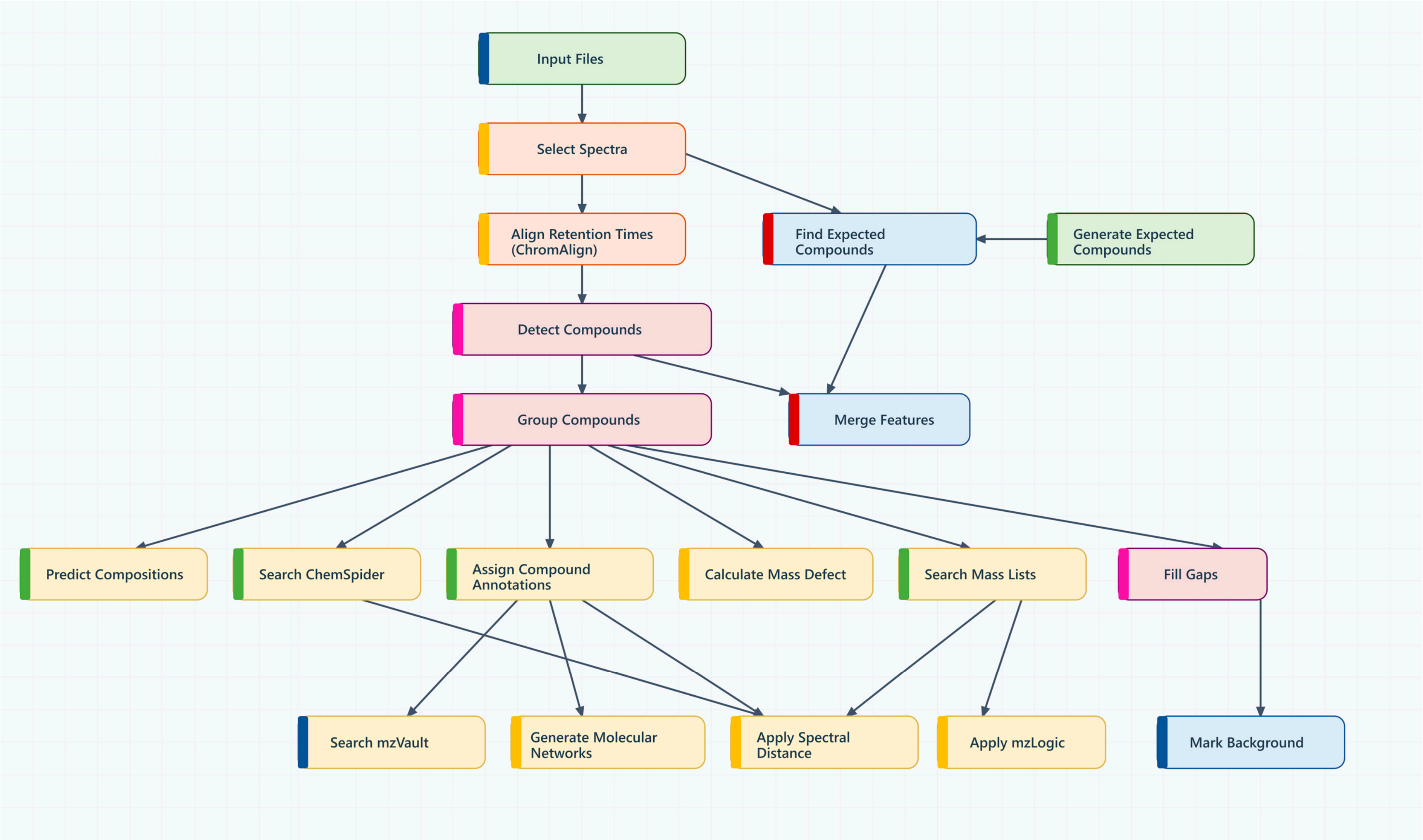

Figure S 4 - Compound Discoverer Analysis Workflow

Compound Discoverer Filter

Table S 1 – Filter applied to Compound Discoverer features

Parameter/Property	Condition/Operator	Value	Context
**Annot. ΔMass [ppm]**	is between	-3.00 and 3.00	—
**P-value**	is less than or equal to	0.05	in any ratio
**Group CV [%]**	is less than or equal to	15.00	in sample group CUAI0124N_AqC
**MS2**	is equal to	DDA for preferred ion	—
**Annot. Source**	has status	Full match	in at least 2 sources
**Ratio**	is greater than	5.00	in sample group (SAMPLE GROUP) / (BLANK)
**Group Areas**	is rounded greater than or equal to	1000000.00.00	in sample group SAMPLE GROUP
**Annot. Source**	has at least status	Full match	in source mzCloud Search
**Peak Rating**	is rounded greater than or equal to	6.00	in sample REPLICA1.raw AND REPLICA2.raw AND REPLICA3.raw

Microplastics Acronyms

Table S 2 – SMPs acronyms and their respective polymer name

Acronyms	Polymer name
Acrylic polymers	Acrylic polymers
Butene Ethylene copolymer	BE copolymer
EVA	Ethylen Vynil Acetate
EVOH	Ethylen Vynil Alchol
HDPE	High Density Polyethylene
LDPE	Low Density Polyethylene
OLEFIN	OLEFIN
PA6	Polyamide 6
PDMS	Polydimethylsiloxane
PEAA-Zn	Zinc salt of ethylene acrylic acid copolymer
PE-PAA	PB/EC
PES	Polyester
POM	Polyoximethylene
PP	Polypropylene
PP+ PE copolymer	Polypropylene and Polyethlene Copolymer
PS	Polystyrene
PTFE	Polytetrafluoroethylene
PVC	Polyvynilchloride
PVE	Polyvynil ethers

Best Micro-FTIR Spectra

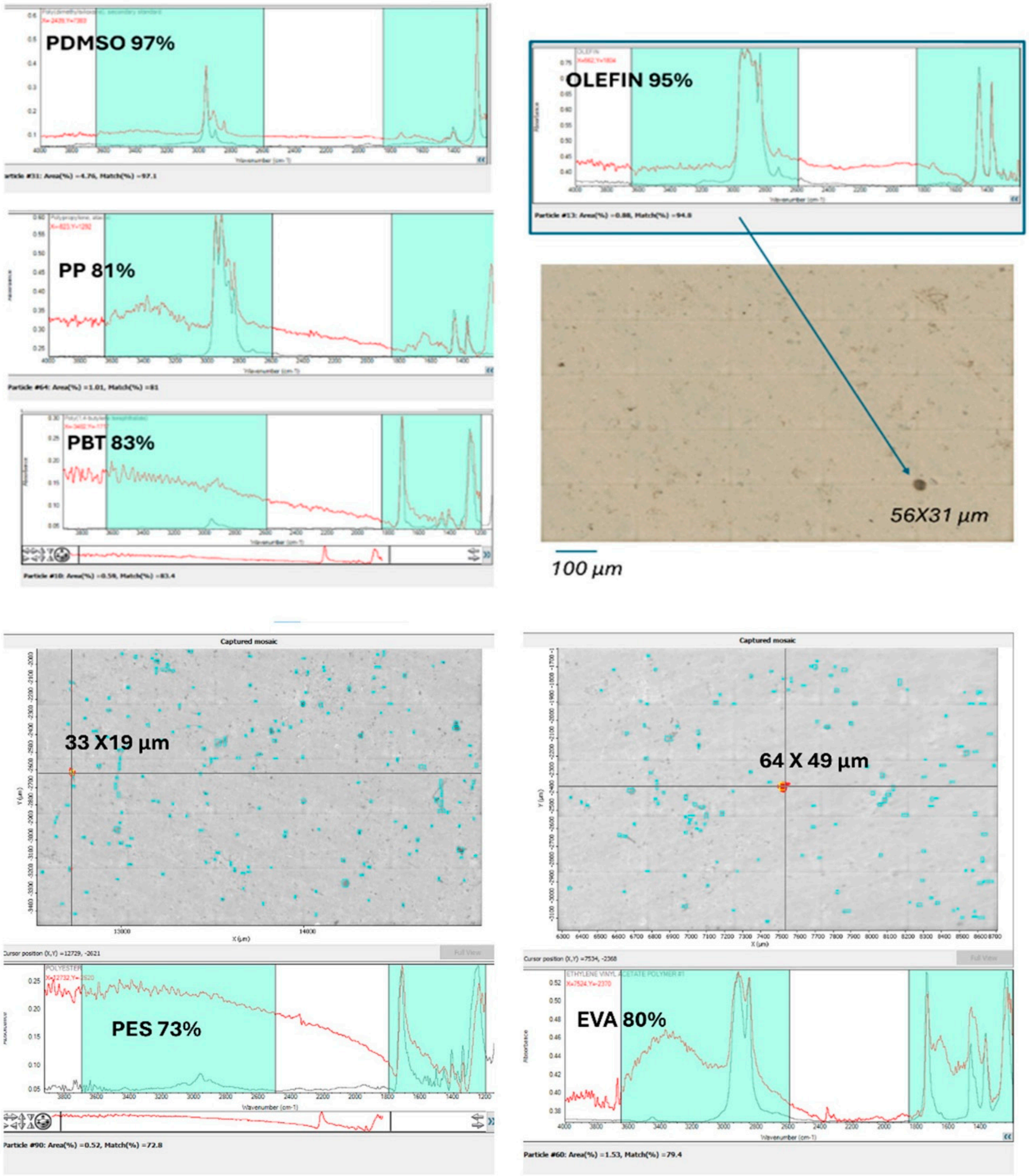

Figure S 5 - Best spectra collected in the samples of freshwater analyzed during the sampling period under exam

MLCs reported as classes of compounds

Table S 3 – Abundance of MLCs L-1 for each month grouped in classes of compounds

	06/03/23	04/04/23	08/05/23	06/06/23	04/07/23	31/07/23	12/09/23	02/10/23	13/11/23	11/12/23	10/01/24	13/02/24
**Pigments**	542	903	1807	1265	1084	903	723	3252	723	903	181	903
**Antioxidants**		361	2529	723	361	1084				1626		
**Light stabilizer**	1084	181	1084	903		1265	181			994	542	
**Filler**	2890	181	3252	1445		2710	1987	1265	1987	1987		1084
**Natural fiber**	1987	1806	3071	2890	361	5781	723	903	542	3071	361	
**Semi-synthetic fiber**	181	181	542	542						361		
**Plasticizer**			181									
**Antistatic agent**	1445		723	723		1265	6684	6323	6684	1626	5781	3974
**Colorants**	181	2529	361	361	3974	723	361	2168	542	903	723	1084
**Lubricants and plasticizer***		181	181	181		361	2168	2529	2529	361	3794	1987
**Lubricant**		181							181	361		
**Coating**		181		1626	361	903			361	903	181	
**Biopolymer**	181			723		181		1265	181	181	361	361
**Vulcanizer**					181	181			361	181	361	
**Curing agents**									181	181		
**Cross-linking agent**							181		181			
**Total**	**8491**	**8310**	**13729**	**11381**	**6323**	**15355**	**13007**	**17704**	**14452**	**13639**	**12284**	**9394**

*MLCs that are both plasticizers and lubricants

MPs size distribution

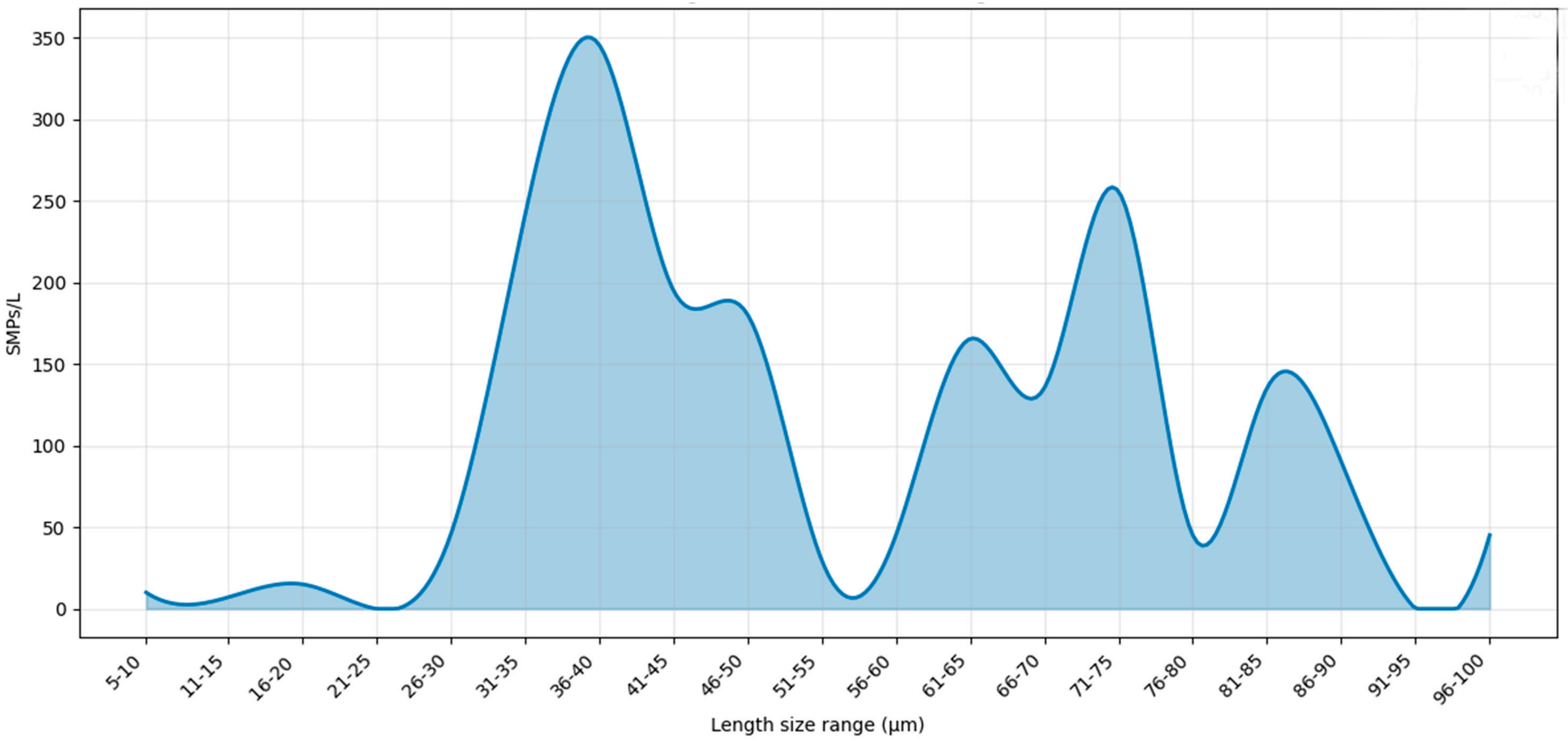

Figure S 6 - Size (length, µm,) distribution of MPs in all the samples under study

MLCs Size distribution

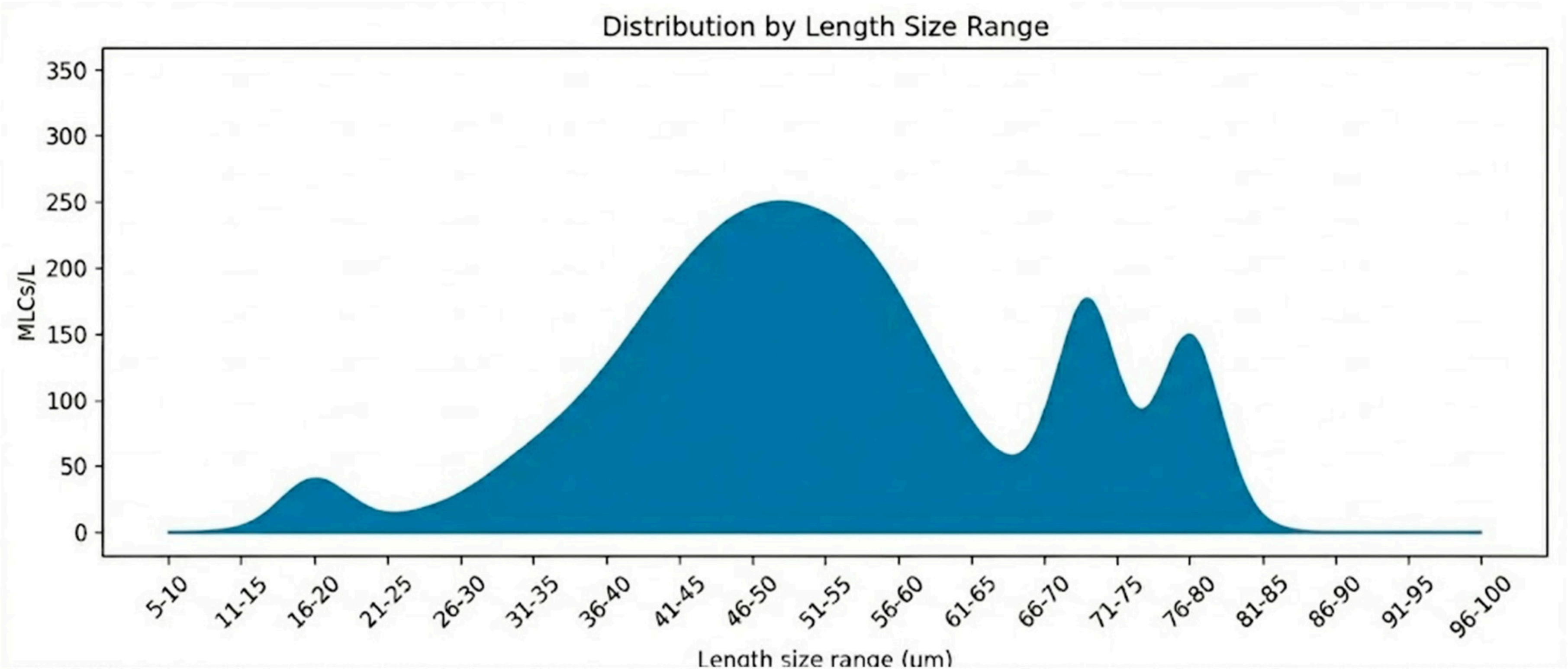

Figure S 7 - Size (length, μm,) distribution of MLCs in all the samples under study

Collection of Best HRMS Spectrta

a)

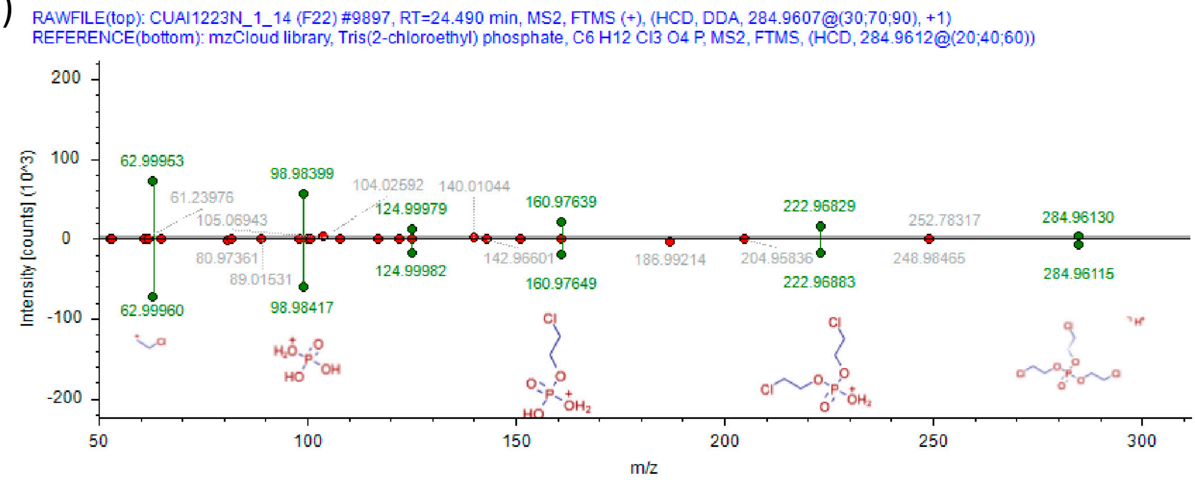

b)

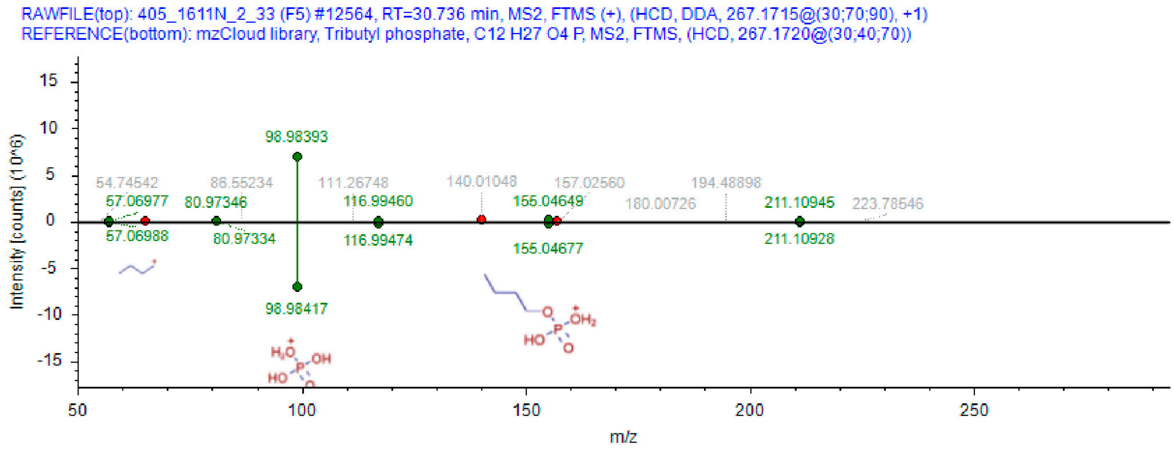

c)

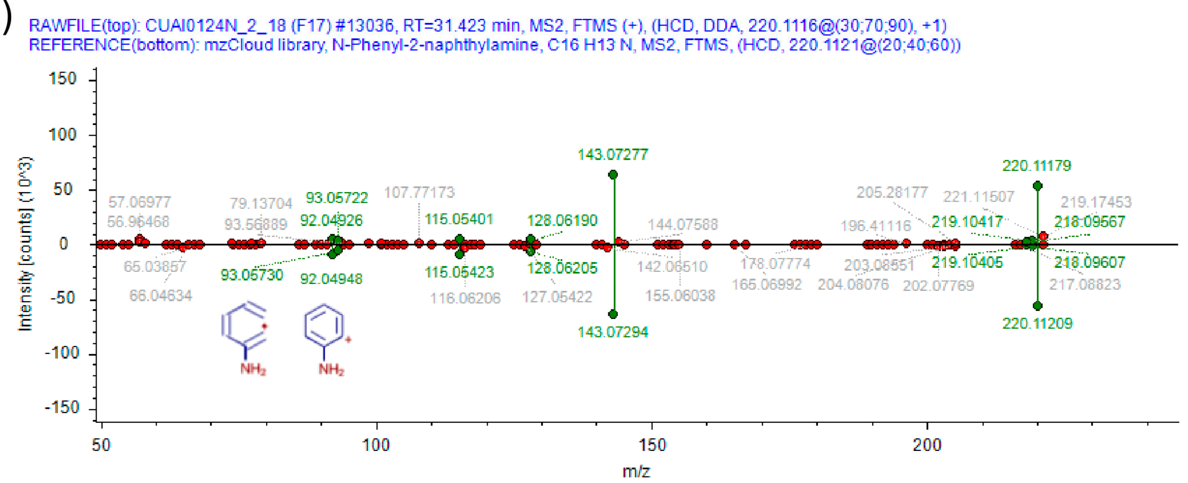

Figure S 8– Selection of matching spectra. Top: rawfile, bottom: mzCloud reference. a) Tris(2-chloroethyl)phospahte; b) Tributyil phosphate; c) N-Phenyl-2-naphthylamine

a)

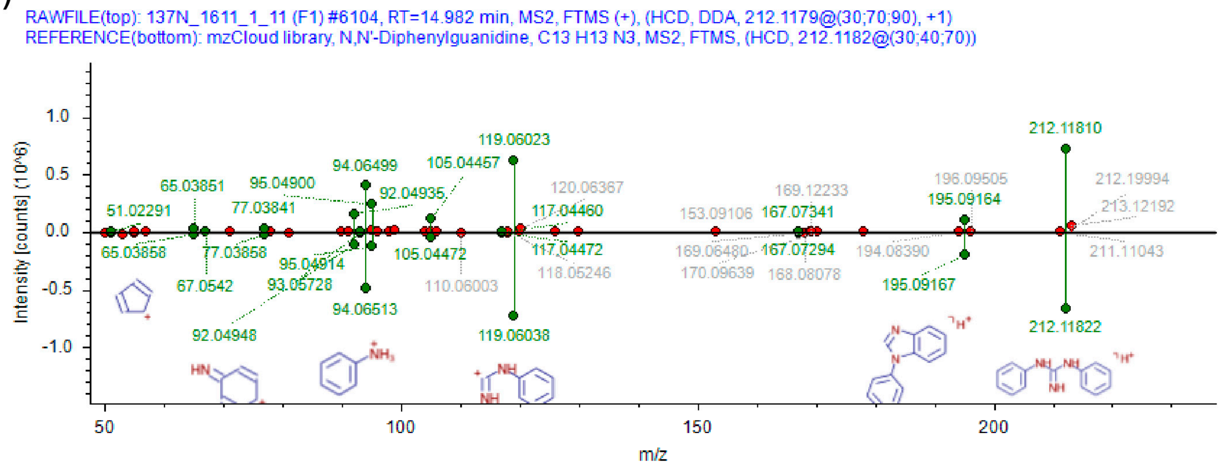

b)

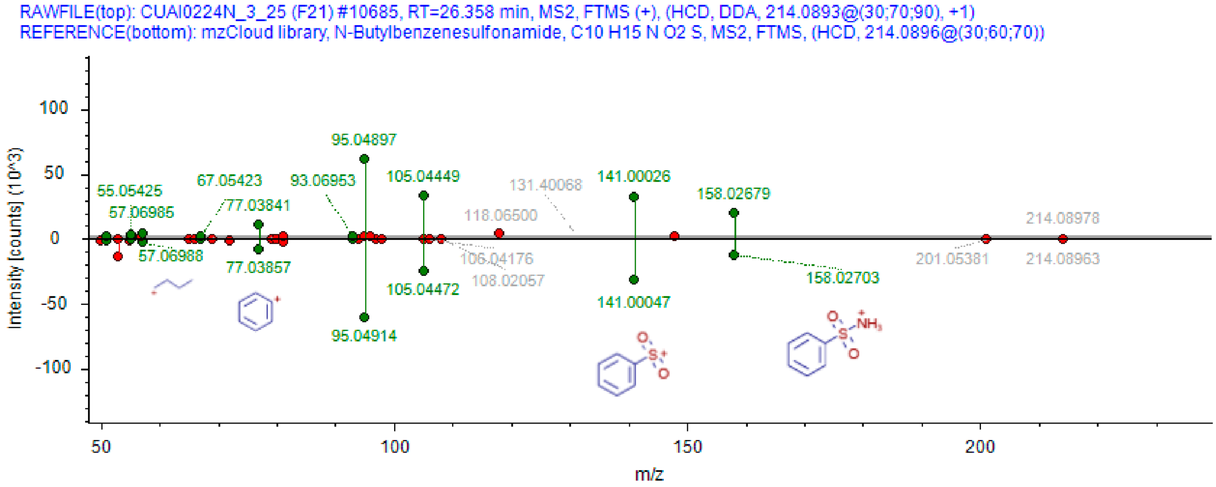

c)

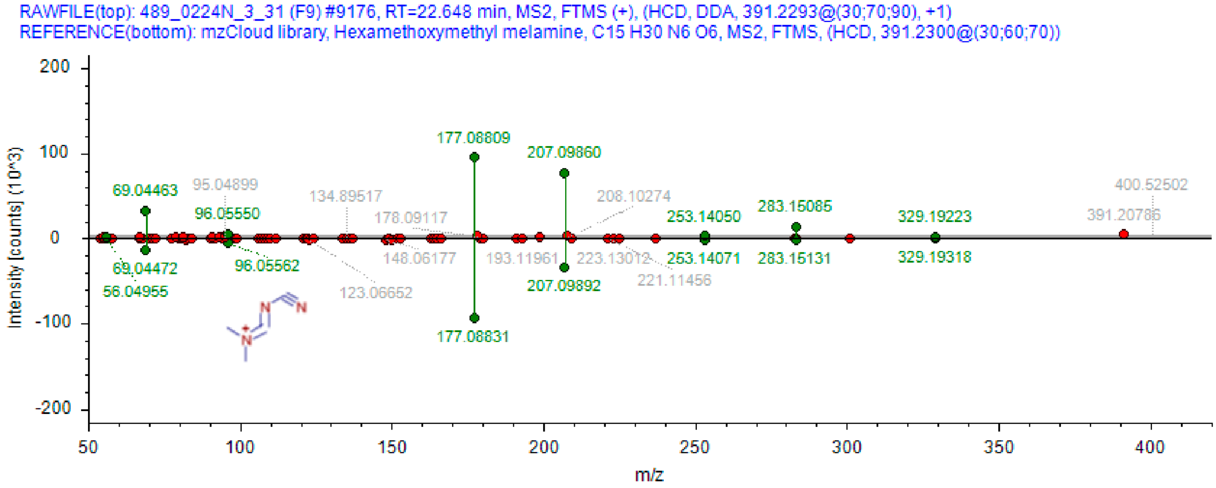

Figure S 9 – Selection of matching spectra. Top: rawfile, bottom: mzCloud reference. a) N,N′-Diphenylguanidine; b) N-Butylbenzenesulfonamide; c) Hexamethoxymethyl melamine

a)

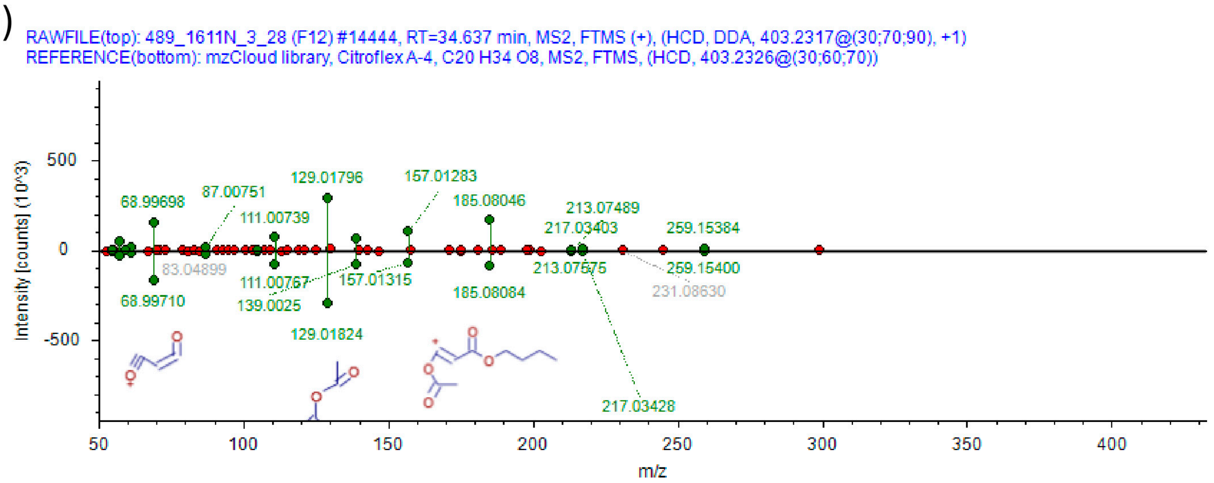

b)

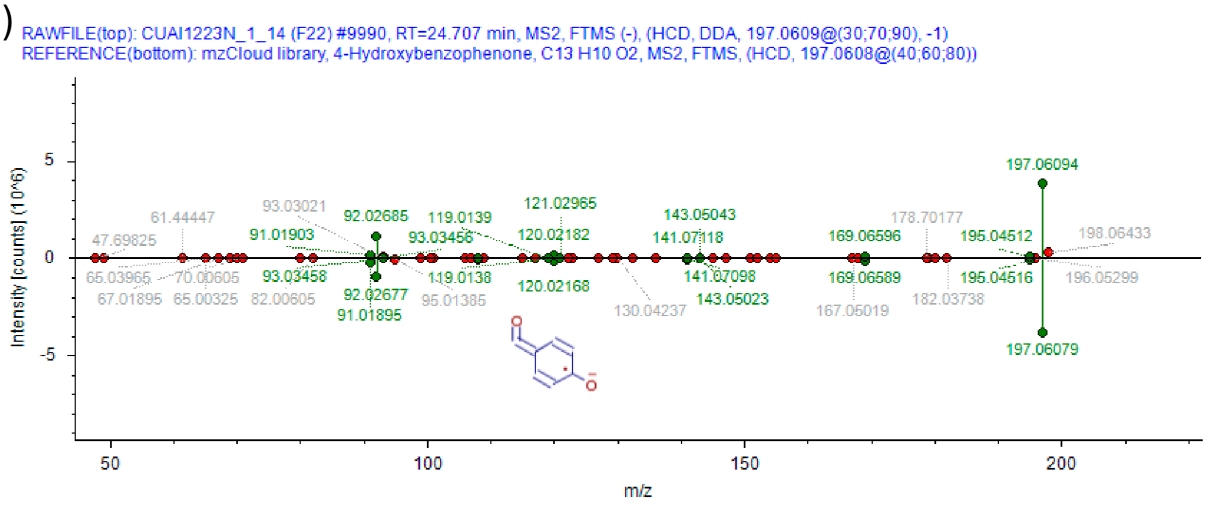

c)

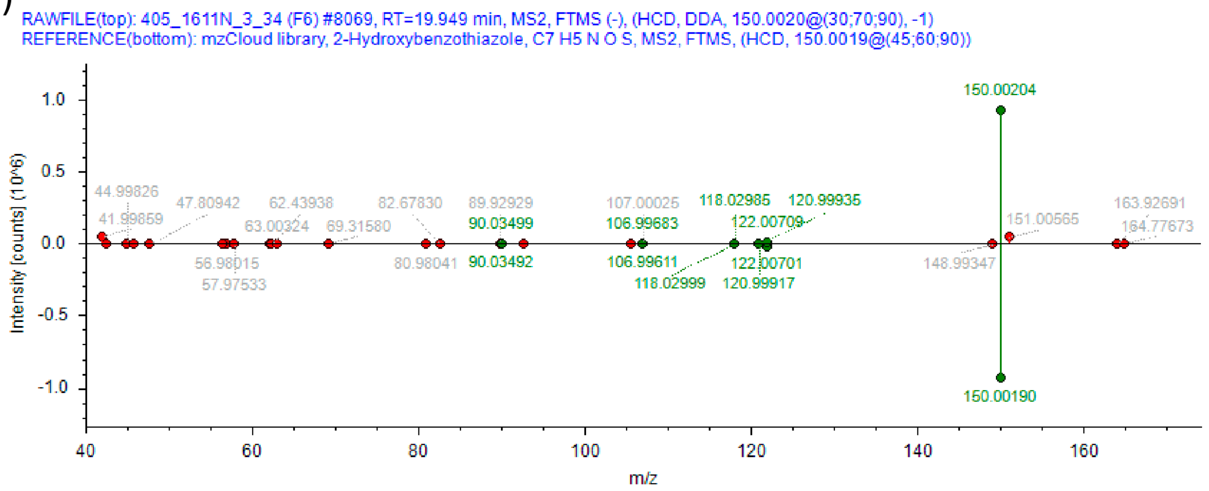

Figure S 10 - Selection of matching spectra. Top: rawfile, bottom: mzCloud reference. a) Citroflex A-4 b) 4-Hydroxybenzophenone; c) 2-Hydroxybenzothiazole

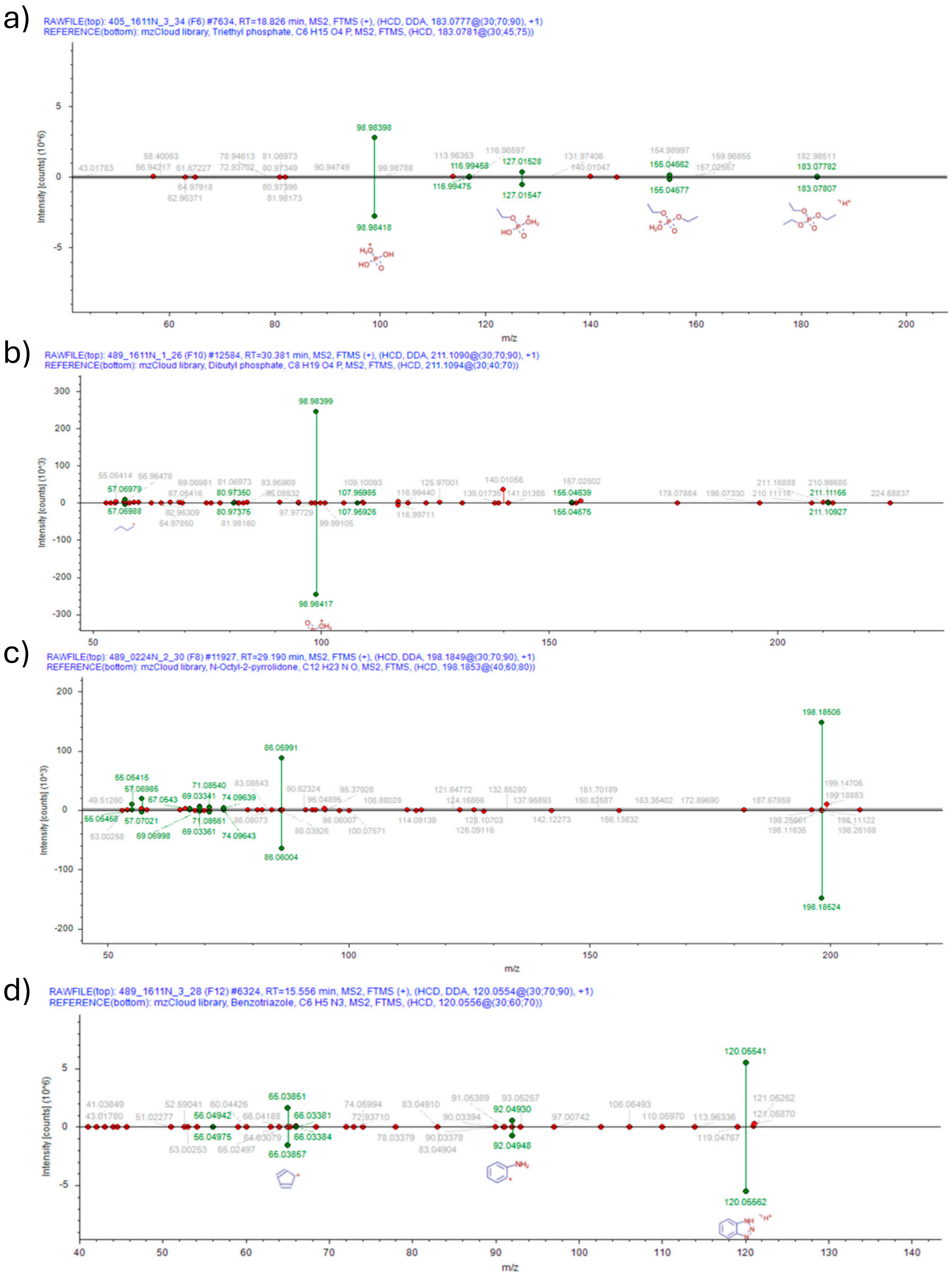

Figure S 11 - Selection of matching spectra. Top: rawfile, bottom: mzCloud reference. a) Triethyl phosphate; b) Dibutyl phosphate; c) N-Octyl-2-pyrrolidone; d) Benzotriazole

Matching with reference standards

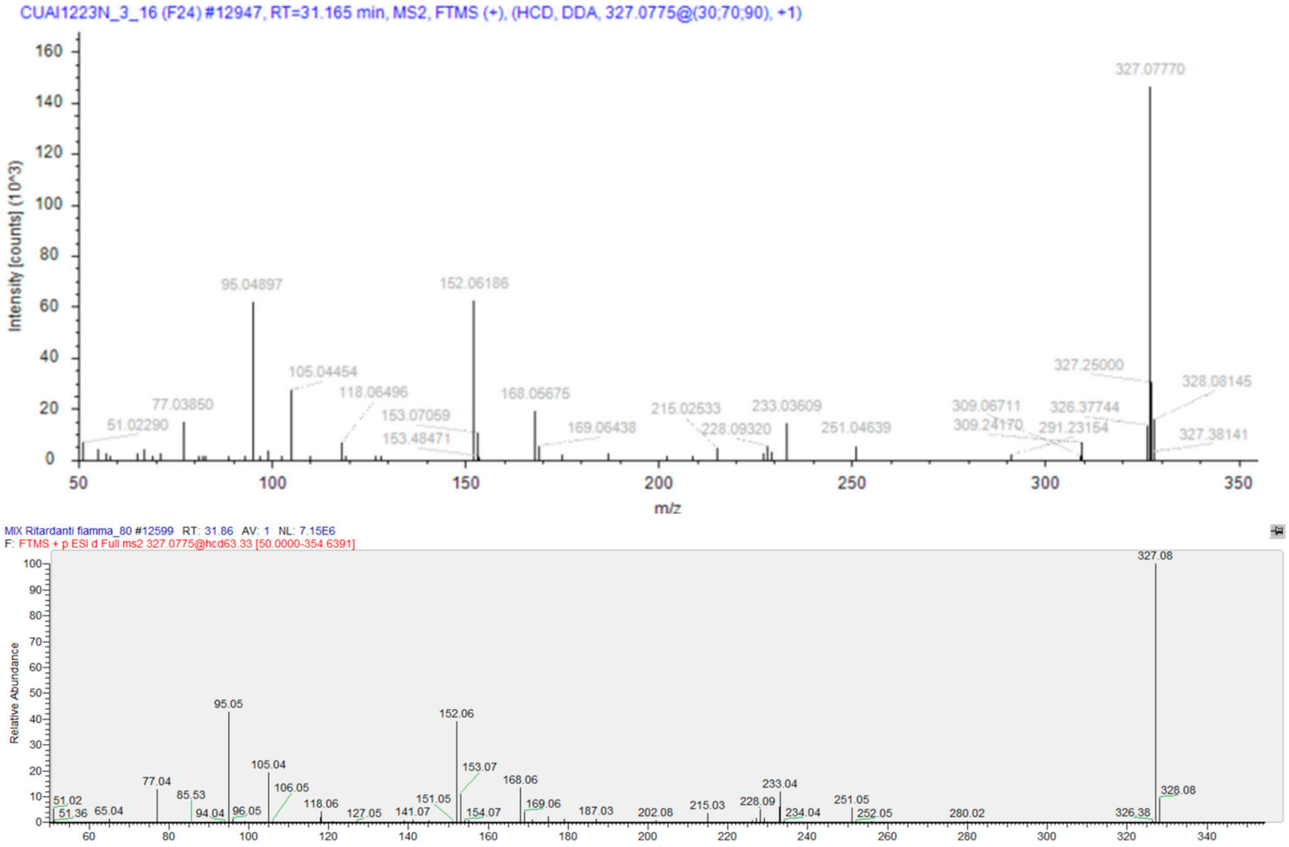

Figure S 12 High-resolution MS^2 spectral comparison for **Triphenyl phosphate** (m/z 327.0775, $[M+H]^+$), in ESI+ mode. **Top:** MS^2 spectrum of the analyte detected in the sample. **Bottom:** MS^2 reference spectrum of the analytical standard (RT = 31.86 min). Fragmentation was obtained using HCD. Main diagnostic product ions (e.g., m/z 77.04, 95.05, 105.04, 152.06, 168.06, 233.04, 327.08) are labeled.

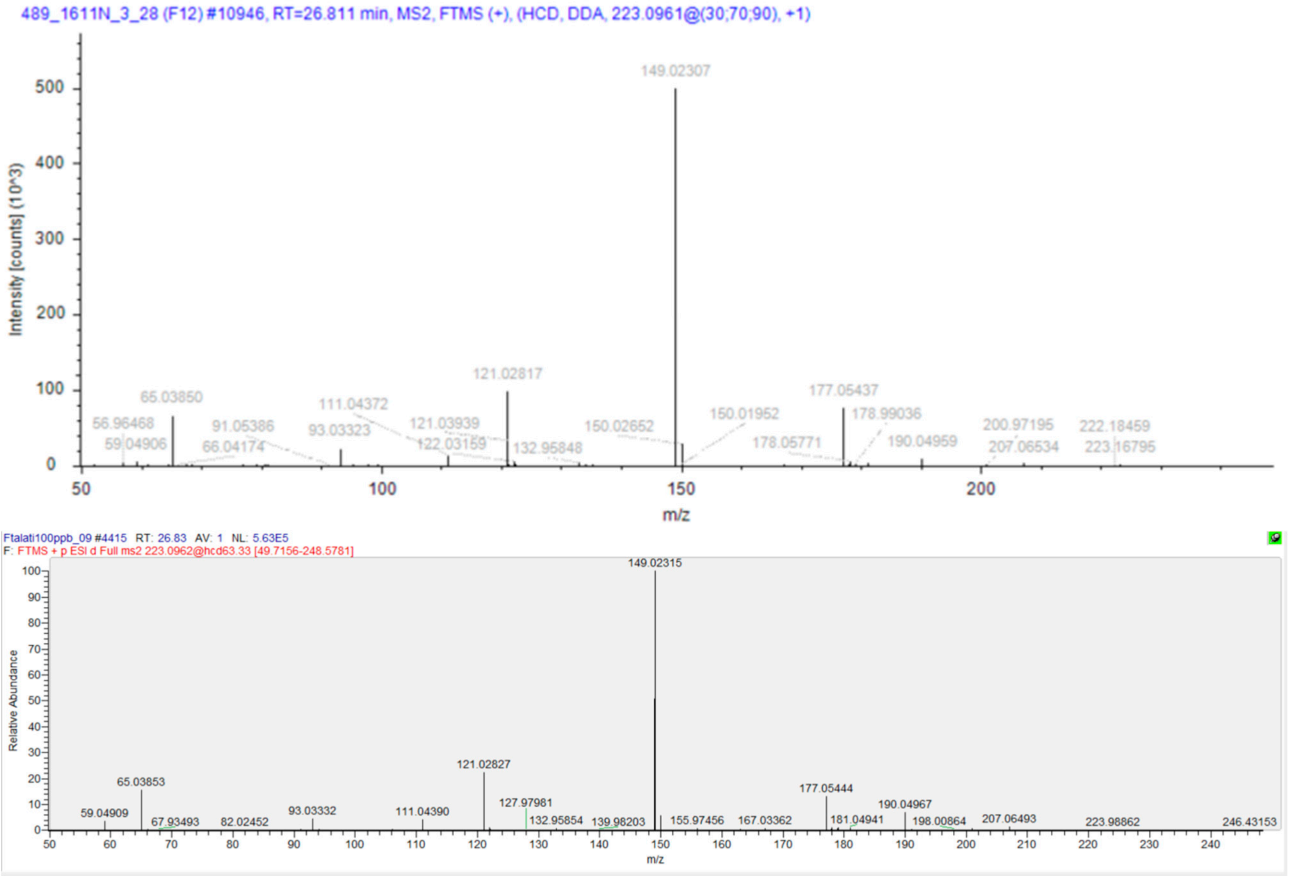

Figure S 13 High-resolution MS^2 spectral comparison for Diethyl phthalate (m/z 223.0961, $[M+H]^+$) in ESI+ mode. Top: MS^2 spectrum of the analyte detected in the sample (RT = 26.81 min). Bottom: MS^2 reference spectrum of the analytical standard (RT = 26.83 min). Fragmentation was obtained using HCD. Main diagnostic product ions (e.g., m/z 65.04, 121.03, 149.02, 177.05) are labeled.

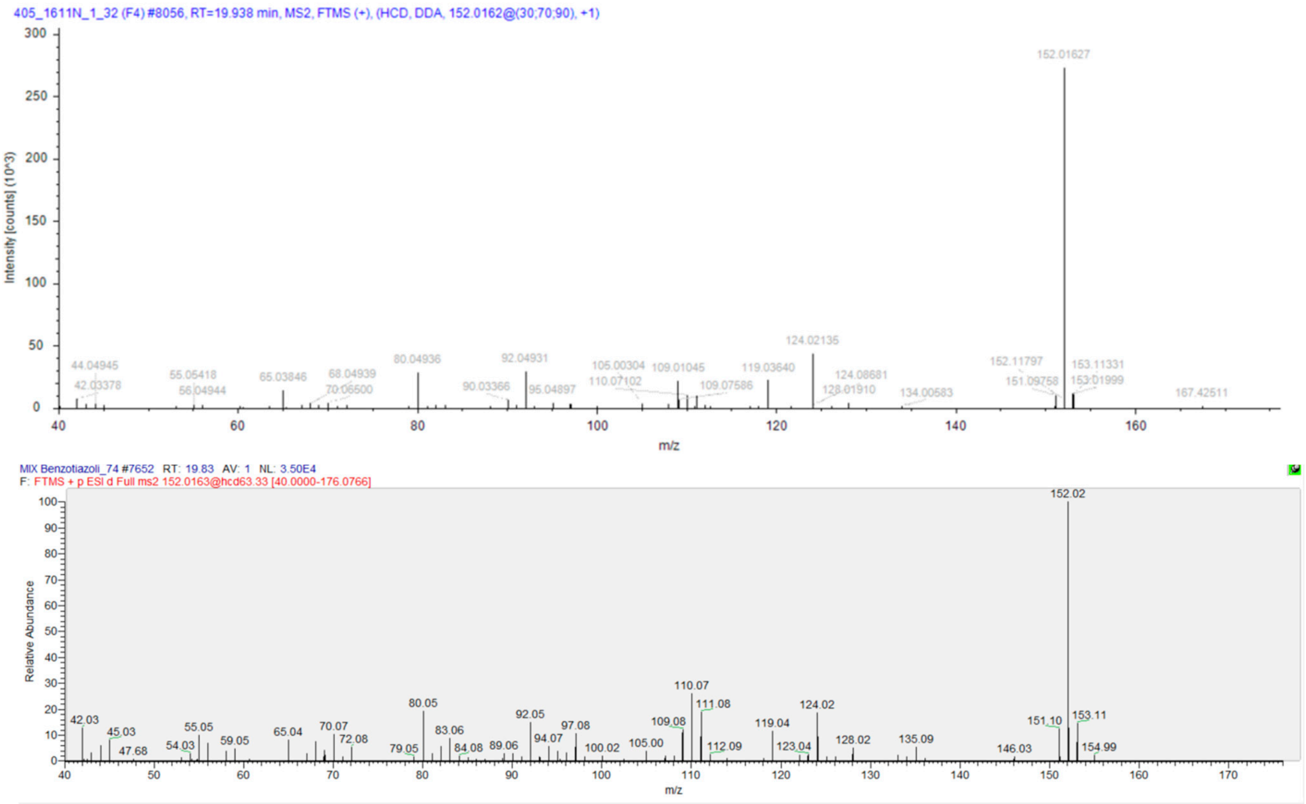

Figure S 14 High-resolution MS^2 spectral comparison for 2-Hydroxybenzothiazole (m/z 152.0162 $[M+H]^+$) in ESI+ mode. Top: MS^2 spectrum of the analyte detected in the sample ($RT = 19.94$ min). Bottom: MS^2 reference spectrum of the analytical standard ($RT = 19.83$ min). Fragmentation was obtained using HCD. Main diagnostic product ions (e.g., m/z 80.05, 92.05, 110.07, 124.02, 152.02) are labeled
